# Current Evidence from Animal Models on Molecular Changes Underlying Antidepressant Effects of Psychobiotics

**DOI:** 10.3390/pharmaceutics18010140

**Published:** 2026-01-22

**Authors:** Nevena Todorović Vukotić, Neda Đorđević, Andrijana Stanisavljević Ilić, Svetlana Soković Bajić, Ivana Perić

**Affiliations:** 1 Department of Molecular Biology and Endocrinology, “Vinča” Institute of Nuclear Sciences—National Institute of the Republic of Serbia, University of Belgrade, 11000 Belgrade, Serbia; neda@vin.bg.ac.rs (N.Đ.); andristanisavljevic@vin.bg.ac.rs (A.S.I.); ivanap@vin.bg.ac.rs (I.P.); 2 Group for Probiotics and Microbiota-Host Interaction, Department of Microbiology and Plant Biology, Institute of Molecular Genetics and Genetic Engineering, University of Belgrade, 11000 Belgrade, Serbia; svetlana.sokovic@imgge.bg.ac.rs

**Keywords:** psychobiotic, probiotic, depression, animal model, behavior, HPA axis, neuroinflammation, neurotransmission, tryptophan metabolism

## Abstract

The treatment of depression is an uphill battle due to the low efficiency and delayed clinical response of antidepressants and the fact that most of them cause numerous side effects. Psychobiotics, probiotics that affect brain function and confer mental health benefits, emerged as a promising ally showing protective effects against depressive- and anxiety-like behaviors in various animal models of depression. There is rapidly accumulating evidence that psychobiotics show protective effects at the molecular level as well, affecting several pathophysiological processes implicated in depression. This narrative review summarizes preclinical insights into molecular changes related to the hypothalamic-pituitary-adrenal (HPA) axis, peripheral inflammation, neuroinflammation, neurotransmission and tryptophan metabolism underlying psychobiotic-driven mitigation of depressive and anxiety symptoms in stress-based, corticosterone-induced and inflammation-induced animal models of depression. Research evidence indicates that psychobiotics normalize the activity of the HPA axis, decrease levels of inflammatory mediators in the intestine, circulation, and brain, normalize the levels of neurotransmitters and their receptors, and regulate tryptophan metabolism in various animal models of depression. The main setbacks in this field are the extensive diversity of studied probiotic strains, which are often insufficiently characterized, and the lack of mechanistic studies in animal models. However, despite these challenges, further study of psychobiotics in the pursuit of supportive therapies for depressive disorders is firmly grounded.

## 1. Introduction

Patients with depressive disorder in the acute phase undergo pharmacotherapy, in addition to psychotherapy; however, the majority (around 60%) do not benefit from their first antidepressant [1]. In addition, achieving remission and recovering from an acute episode is not enough in terms of the treatment goal, given the very high risk of relapse and recurrence [2,3]. Maintenance therapy is recommended for patients with recurrent depression who have achieved recovery [4]. However, antidepressants often produce side effects, including weight gain, sexual dysfunction, drowsiness, insomnia, anxiety, dizziness, headache, dry mouth, blurred vision, nausea, rash, and tremor [5], and they also carry a risk of hepatotoxicity [6]. The exploding rates of depression, low efficacy, and delayed action of antidepressants give high priority to the improvement of antidepressant therapy and reflect the need for combinations of treatments for maximum clinical benefit. In order to be utilized for the prevention of relapse and recurrence in patients, novel therapeutic strategies should be safe for long-term use. The scientific community has put a lot of effort over the last decade into finding new agents with antidepressant activity, mainly among natural products. Following numerous preclinical and clinical studies highlighting the role of compositional and functional alterations in the gut microbiota (dysbiosis) in the onset and progression of depression, with evidence showing that restoring microbial balance exerts antidepressant-like effects, microbiota-based therapeutics have emerged as a promising treatment strategy for depression [7]. The gut microbiota communicates with the central nervous system (CNS) and affects mood and behavior by producing or stimulating the production of various neurotransmitters (serotonin-5-hydroxytriptamine-5-HT; gamma-aminobutyric acid-GABA; dopamine-DA; noradrenaline-NA), short-chain fatty acids (SCFAs), and hormones, among others [7]. Gut microbiota and the CNS, together with the signals they exchange through neural, endocrine, immune, and humoral pathways, are known as the microbiota-gut-brain-axis (MGBA). Recent cultivation-based work has demonstrated that numerous anaerobic gut strains possess immunomodulatory activity and produce metabolites relevant to gut–brain communication [8]. Moreover, work on poorly characterized *Bacteroides* species has demonstrated that certain isolates can reduce intestinal and peripheral inflammatory signaling and modulate GABA-, 5-HT-, and dopamine-related gene expression, further supporting their potential role in the MGBA [9]. The identification of depression-associated microbial taxa remains an active area of research. Recent meta-analyses and microbiome studies consistently report alterations in bacterial groups linked to key metabolites implicated in depression, including butyrate, a major anti-inflammatory short-chain fatty acid (*Faecalibacterium*, *Coprococcus*, *Subdoligranulum*, *Butyricicoccus*, *Eubacterium*, and *Fusicatenibacter*), and those involved in tryptophan (Trp)/5-HT, glutamate and GABA metabolism (*Lactobacillus* spp., *Bifidobacterium* spp., and *Akkermansiaceae*) [7,10,11]. Conversely, an enrichment of potentially pro-inflammatory taxa such as *Eggerthella* and *Flavonifractor* has been reported. Based on these findings, two main strategies have emerged in psychobiotic research: (1) restoring depleted beneficial taxa, and (2) supplementing strains with proven capacities to produce neuroactive and immunomodulatory metabolites such as GABA, serotonin precursors, and SCFAs. Clinical trials published so far, although limited by small sample sizes and narrow assessments of dosage and individual probiotic strain effects, indicate that probiotics may be efficacious as an adjunct or even as a standalone therapy for treating major depressive disorder [12,13,14,15]. In light of all the aforementioned promising findings, one may reasonably conclude that psychobiotics, the emerging group of probiotics that affect brain function and confer mental health benefits, might revolutionize the therapeutic approach to mood disorder alleviation [16]. Psychobiotics include not only live microorganisms, but also parabiotics (inactivated, nonviable intact microbial cells) and postbiotics (metabolic products secreted by probiotics or released after bacterial lysis), which exert a beneficial effect on the brain. Growing evidence suggests that psychobiotics can ameliorate several pathophysiological aspects of depression, including hypothalamic-pituitary-adrenal (HPA) axis dysfunction, peripheral inflammation, neuroinflammation and disturbed neurotransmission. There is also strong evidence that psychobiotics may affect changes in Trp metabolism associated with depression and therefore modulate 5-HT synthesis [17]. While numerous research studies have solidified evidence of the antidepressant potential of psychobiotics, precise molecular events still await comprehensive elucidation. Herein, we summarize the antidepressant effects that psychobiotics have shown in animal models of depression, and review molecular changes related to the HPA axis, peripheral inflammation, neuroinflammation, neurotransmission and Trp metabolism underlying those effects.

## 2. Methodology of the Review

We conducted a narrative review using a structured literature search strategy. PubMed, Scopus, and Science Direct databases were comprehensively searched for original articles using the search terms “anhedonia”, “anxiety”, “HPA axis”, “inflammation”, “neuroinflammation”, “neurotransmitters”, “tryptophan metabolism” cross-referenced with “depression” and “psychobiotics”/”probiotics” from inception until 31 August 2025. This review included only studies published in English. Studies involving symbiotics or probiotics with bulking agents were excluded from the analysis due to the inability to determine the effect of the probiotic alone. The selected publications were organized according to the listed thematic categories. A tabular overview of the experimental findings regarding the effects of probiotics in animal models of depression within each category was provided.

## 3. Depressive- and Anxiety-like Behavior

Numerous studies have reported that psychobiotic treatment prevented or ameliorated behavioral changes in animal models of depression. Table 1 summarizes experimental studies investigating the effects of psychobiotics on depressive- and anxiety-like behaviors. A detailed overview of behavioral parameters indicating antidepressant and anxiolytic effects of psychobiotics can be found in Table S1 (Supplementary Material). Bacterial strains from the genera *Lactobacillus* and *Bifidobacterium* were the most commonly used as psychobiotics, while chronic unpredictable mild stress (CUMS) was the most frequently used protocol for inducing a depression-like phenotype. Depression-like behavior was monitored mainly by assessing behavioral despair using the forced swim test and the tail suspension test, anhedonia using the sucrose preference test, and social behavior using the social interaction test. In addition, the majority of studies also monitored anxiety by the open field test, the elevated plus maze, the elevated zero maze and the light-dark box test. Most studies reported protective effects against both anhedonia and anxiety. However, Liu et al. [18] found that *Lactobacillus plantarum* PS128 reversed depression-like, but not anxiety-like, behavior in maternal separated mice. Only a few studies have addressed the question of sex-specific effects of psychobiotics. Satti et al. [19] and Dandekar et al. [20] found that psychobiotics prevented anxiety- and depression-like behaviors in both male and female rats, while Carlessi et al. [21] found sex differences in the efficacy of *Bifidobacterium infanti.* Namely, *B. infanti* was shown to protect against depressive-like behavior in female, but not male, rats. The available findings are inconsistent, highlighting the need for more studies including both sexes to better understand potential sex-specific effects of psychobiotic actions.

In addition to having protective effects in stress-based models of depression, psychobiotics also reversed behavioral changes in corticosterone [32], lipopolysaccharide (LPS)- [53] and alcohol-LPS-induced [58] models. Interestingly, the latest study reported antidepressant and anxiolytic effects of a probiotic in a depression model induced by transplantation of cultured fecal microbiota from patients with depression [26]. Furthermore, psychobiotics have been shown to exert beneficial effects on behavioral changes associated with other states, both physiological, like aging, and pathological, like nerve injuries or infections. Gao et al. [68] showed that two strains within the *Lactococcus* genus decreased depression- and anxiety-like behaviors in aged mice. Also, a mixed-strain culture of *Bifidobacteria* and *Lactobacilli* was found to alleviate anxiety-like behavior associated with brachial plexus avulsion [69]. Furthermore, *Lactiplantibacillus plantarum* RTA8 has been reported to ameliorate behavioral abnormalities related to *Salmonella* infection in female mice [70]. Interestingly, *Bacillus amyloliquefaciens* SC06 attenuated anxiety-like behavior in high-fat diet-fed male mice [71], whereas a probiotic mixture consisting of eight bacterial strains (mainly strains of *Lactobacillus* and *Bifidobacterium*) protected high-fat diet-fed rats from anhedonia [72]. Finally, a psychobiotic mixture containing *Lactobacillus plantarum* 90sk and *Bifidobacterium adolescentis* 150 decreased immobility time in the forced swim test, an indicator of depressive-like behavior, compared to control untreated mice [73].

## 4. HPA Axis

Psychobiotics have been shown to be protective on behavior, and several molecular mechanisms underlying their actions have been reported to date. Growing lines of evidence suggest that psychobiotics ameliorate stress-induced changes in HPA axis functioning (Table 2).

Plasma or serum levels of CORT, one of the most extensively studied hormones related to stress, were also the most commonly monitored stress-related endocrinological parameter in psychobiotic studies. The vast majority of studies have found that treatment with probiotics ameliorated stress-induced increase in plasma/serum CORT levels. These effects appeared to be independent of psychobiotic treatment duration. While a 2-week treatment with *Lactiplantibacillus plantarum* D-9 in mice exposed to CUMS resulted in a decline in serum CORT [23], a comparable 2-week treatment with *Mycobacterium vaccae* in rats showed no effect [78]. These discrepancies may be attributable to differences in animal models and/or the specific psychobiotic strains employed. Notably, the majority of studies reporting undefined or inconsistent CORT alterations were conducted in mice. Interestingly, the probiotics *Lactococcus lactis* ZFM559, *Bifidobacterium longum* CECT 30763, Lpc-37 and LP12407 alleviated depressive- and/or anxiety-like behaviors despite having no effect on CORT levels [31,52,56]. A recent study reported that administration of *Bifidobacterium longum* CECT 30763 attenuated adrenal gland atrophy in mice subjected to chronic social defeat stress (CDSD) [51]. Ait-Belgnaoui et al. showed, more than a decade ago, that *Lactobacillus farciminis* attenuated the HPA response to an acute psychological stress in rats by preventing gut leakiness and a consequent increase in circulating LPS levels [79]. Recent studies corroborated the finding that probiotic treatment ameliorated stress-induced changes in HPA axis functioning. Jia et al. found that *Lactiplantibacillus plantarum* D-9 prevented a CUMS-induced increase in hypothalamic corticotropin-releasing hormone (CRH) and serum CORT levels in mice [23]. Consistently, Ma et al. reported that *Lactobacillus plantarum* GM11 prevented an increase in hippocampal (HIPP) and serum CORT levels in a CUMS model [33]. Interestingly, *Bifidobacterium breve* CCFM1025 not only prevented a CUMS-induced increase in serum CORT and hypothalamic CRH levels, but also normalized expression of *Nr3c1*, a gene that encodes the glucocorticoid receptor (GR), in HIPP [47]. Namely, Tian et al. reported that CUMS impaired the negative feedback of CORT in the HPA axis by downregulating the GR in HIPP, which can lead to glucocorticoid resistance. So, CCFM1025-driven increase in GR expression, restoration of impaired CORT negative feedback, and alleviation of glucocorticoid resistance are behind mitigation of depression-like behavior observed in the case of this probiotic strain [47]. In contrast, Tiwari et al. recently reported that *Lactobacillus fermentum* ATCC 9338 prevented depression-like behavior in CUMS mice through decreasing GR expression [76]. Namely, they found that CUMS mice exhibited increased GR expression in the cortex, HIPP, and medulla, while supplementation with ATCC 9338 significantly reduced GR expression, which, according to the authors’ speculations, suggested a protective role of the used probiotic against stress-induced neurodegeneration. This contradiction in GR expression found in the depression model is even more surprising considering that both studies used CUMS for modeling depression in male mice. However, it should be noted that Tian et al. subjected mice to a 6-week-long CUMS model [47], while Tiwari et al. used a 4-week-long CUMS model of depression [76]. Thus, the difference in the duration of stress exposure may be responsible for the noted discrepancies. Even more captivating is the fact that in both cases, probiotic treatment normalized the expression of GR in the depression model, despite the opposite effects induced by CUMS. Interestingly, two different probiotic strains (*Bifidobacterium breve* CCFM1025 vs. *Lactobacillus fermentum* ATCC 9338) that were administered for different periods of time (5 weeks vs. 4 weeks) exhibited contrasting effects; nevertheless, each strain led to normalization of GR expression. Regarding CRH, consistent changes in hypothalamic and serum levels were observed following 2- to 6-week psychobiotic treatments in animal models; however, the overall number of studies reporting CRH outcomes remains scarce. Consequently, additional studies are warranted to clarify the roles of treatment duration and strain-specific psychobiotic effects. It should be noted that several studies have reported a decline in serum or plasma adrenocorticotropic hormone (ACTH) levels following treatment with various strains from the *Lactobacillus* and *Bifidobacterium* genera lasting from 3 to 6 weeks [30,75,77]. An opposite direction of change (i.e., an increase in serum ACTH) was documented in only one study, in which mice were treated with LP12418 for 5 weeks [31]. Interestingly, that study reported a further discrepancy relative to the majority of published findings: the absence of any detectable change in plasma CORT levels.

**Figure 1:**
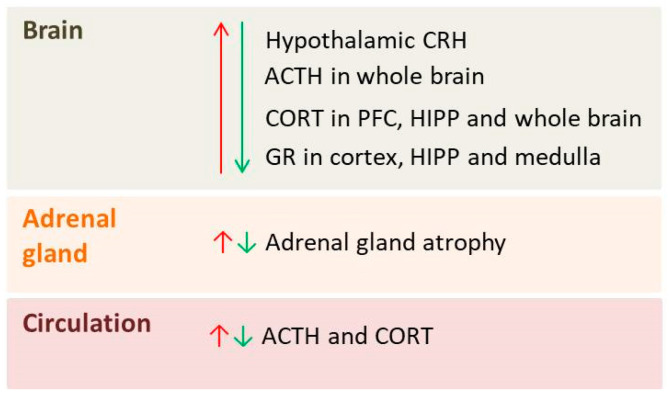
Overview of parameters of the hypothalamic-pituitary-adrenal (HPA) axis affected by depression (red arrows) and psychobiotic treatment (green arrows) in the brain, adrenal gland and circulation: CRH—corticotropin-releasing hormone; ACTH—adrenocorticotropic hormone; CORT—corticosterone; PFC—prefrontal cortex; HIPP—hippocampus; GR—glucocorticoid receptor.

There is also evidence that probiotics prevent depressive behavior by inhibiting HPA-axis hyperactivity in a CORT-induced rat model of depression. Zhao et al. found that administration of *Lacticaseibacillus rhamnosus* TF318 corrected CORT-induced deviations in the gut microbiota by significantly increasing the relative abundance of *Firmicutes*, and normalized HIPP levels of ACTH and CRH, as well as serum levels of CORT, which altogether resulted in the attenuation of depressive-like behaviors [74]. Interestingly, Warda et al. found that 6 weeks of supplementation with a parabiotic of two *Lactobacillus* strains (*Lactobacillus fermentum* and *Lactobacillus delbrueckii*) significantly lowered baseline CORT levels compared to the control rats. Despite the lower baseline levels of CORT, regulation of the HPA axis in response to acute stress (FST) was not affected in treated animals since both control and treated animals followed the same pattern of induction and reduction of the CORT levels [80]. Figure 1 summarizes the main effects of psychobiotics on the HPA axis in animal models of depression.

## 5. Peripheral Inflammation

Dysbiosis, disrupted homeostasis of the gut microenvironment, intestinal barrier dysfunction, and inflammatory responses, altogether referred to as a gut-derived systemic inflammation, are strongly associated with depression [81]. Although findings related to dysbiosis in patients suffering from depression remain inconsistent, an increased abundance of pro-inflammatory strains and decreased presence of anti-inflammatory strains have generally been observed, which further strengthens the case for the inflammatory hypothesis of depression [7]. It is well established that probiotics have various health-promoting effects, including modulation of the immune response. They improve the gut microbial population, increase mucus secretion, prevent the destruction of intestinal epithelial tight junction proteins and consequently intestinal leakage, which altogether blunts activation of the immune system [82]. Numerous studies have shown that various psychobiotics decrease the levels of pro-inflammatory mediators in peripheral tissues like intestine and spleen, as well as in circulation (serum and plasma) in different animal models of depression (Table 3).

Numerous studies reported decreased levels of LPS and pro-inflammatory cytokines, as well as an increase in anti-inflammatory cytokine IL-10, in the circulation in animal models of depression after treatment with various probiotics [23,24,27,28,35,38,39,44,47,51,57,58,61,67] (Table 3). According to the findings summarized in Table 3, there is considerable inconsistency in the reported effects of psychobiotic treatment on serum pro-inflammatory cytokine levels. Changes in the serum interleukin-1β (IL-1β) were reported exclusively following treatment with different *Lactiplantibacillus plantarum* strains, highlighting pronounced strain specificity. While strains GOLD-GUT-HNU082 [24] and GM1l [33] produced no detectable changes in serum IL-1β levels, strains WH021 [28] and R6-3 [27] induced a reduction. In addition to *Lactiplantibacillus plantarum* strains, only the multi-strain probiotic (*Roseburia inulinivorans*, *Bacteroides uniformis*, and *Eubacterium rectale*) decreased serum IL-1β levels in an animal model of depression [67]. More consistent findings were observed for interleukin-6 (IL-6). Several studies examining various probiotic strains reported decreased IL-6 levels in both serum and colon. A notable exception was *Lactiplantibacillus plantarum* GOLD-GUT-HNU082, which showed no effect on serum IL-6 levels [24]. In contrast, results for serum tumor necrosis factor-α (TNF-α) were highly heterogeneous. Reductions in serum TNF-α were reported following treatment with *Lactocaseibacillus rhamnosus* KY16 and HN001, *Lactobacillus zhachilii* HBUAS52074_T_, *Lactobacillus plantarum* MTCC 9510, *Bifidobacterium lactis* HN019, *Bifidobacterium breve* CCFM1025, *Bifidobacterium longum* CECT 30763, and *Akkermansia muciniphila* (Table 3). Conversely, no TNF-α-lowering effects were observed following administration of *Lactiplantibacillus plantarum* RS128 and GM1l, *Lactiplantibacillus plantarum* GOLD-GUT-HNU082 and R6-3, *Lactobacillus helveticus* NS8, or *Lactococcus lactis* ZFM559. Psychobiotic treatments were administered for up to 4 weeks in most studies, whereas two *Lactiplantibacillus plantarum* strains were evaluated following an extended 8-week treatment period. Based on the available evidence, changes in serum TNF-α and IL-1β appear to be predominantly strain-dependent rather than time-dependent. Nevertheless, additional studies explicitly addressing the effects of psychobiotic treatment duration are required to draw final conclusions. The anti-inflammatory effects of several psychobiotics were also associated with an increase in serum levels of the anti-inflammatory cytokine interleukin-10 (IL-10). Only nine studies assessed IL-10 responses. No changes in IL-10 levels were reported following a 4-week treatment with *Lactobacillus zhachilii* HBUAS52074_T_ in mice. In contrast, other psychobiotic strains induced increases in IL-10 levels at the same treatment duration or even shorter intervention periods (3–8 weeks). These findings suggest that IL-10 modulation is primarily strain-specific rather than treatment duration-dependent, although a combined influence of strain characteristics and treatment length cannot be excluded.

Sun et al. recently reported that *Pediococcus acidilactici* CCFM1344 increased the expression of intestinal barrier-related proteins (claudin 1, zonula occludens-1, and occludin 1) and reduced LPS content in the serum of CUMS mice [61]. Tamayo et al. linked behavioral changes in the CSDS model of depression to systemic inflammation, including increases in plasma pro-inflammatory cytokines TNF-α and IL-6 and chemokines CXCL9, CXCL10 and CCL2. Administration of probiotic *B. longum* restored all of these parameters to control levels, indicating an anti-inflammatory action that may be associated with the amelioration of depressive and anxiety-like behaviors [51]. The authors assumed that the immunomodulatory role of *B. longum* was orchestrated from the gut since they observed an increase in regulatory T cell numbers in both the gut and spleen in CSDS rats. A noteworthy observation from this study was also the increase in the total population of macrophages in the spleen of CSDS mice treated with *B. longum*. Although *B. longum* administration elevated the total macrophage population, it concurrently reduced the pro-inflammatory M1 subpopulation, suggesting that *B. longum* probably increases M2-type macrophages, downregulating inflammation.

**Figure 2:**
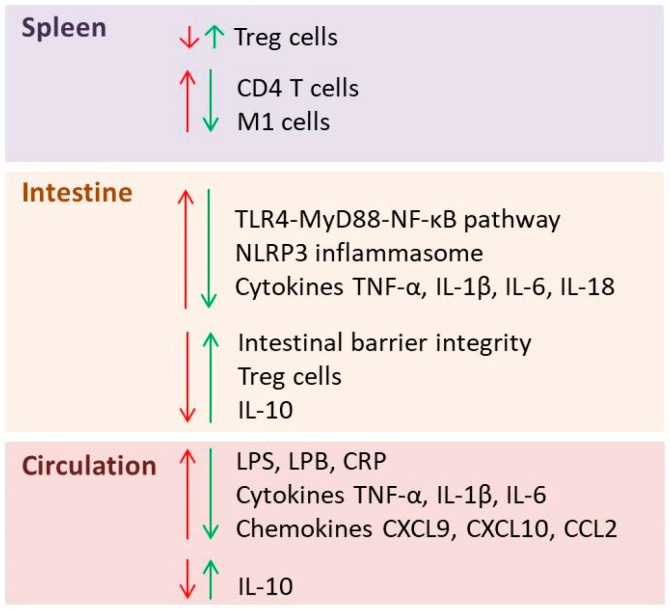
Overview of mediators of inflammation affected by depression (red arrows) and psychobiotic treatment (green arrows) in the spleen, intestine and circulation: Treg cells—regulatory T cells; CD4 T cells—helper T cells; M1 cells—pro-inflammatory macrophages; TLR—Toll-like receptor; MyD88—myeloid differentiation primary response 88; NF-kB—nuclear factor kappa B; NLRP3—NOD-, LRR- and pyrin domain-containing protein 3; TNF-α—tumor necrosis factor-alpha; IL—interleukin; LPS—lipopolysaccharide; LBP—lipopolysaccharide binding protein; CRP—C-reactive protein; CXCL9—chemokine (C-X-C motif) ligand 9; CXCL10—chemokine (C-X-C motif) ligand 10; CCL2—chemokine (C-C motif) ligand 2.

A recently published study reported that *L. plantarum* JYLP-326 enhanced intestinal barrier integrity, inhibited the TLR4-MyD88-NF-κB pathway and decreased the levels of pro-inflammatory cytokines TNF-α, IL-1β and IL-6 in colon, in parallel with alleviating behaviors associated with depression in CUMS mice [25]. Huang et al. demonstrated that a two-strain probiotic containing *L. rhamnosus* HN001 and *B. animalis* subsp. *lactis* HN019 inhibited the activation of NLRP3 inflammasome in colon and improved depressive-like behavior in CUMS rats [83]. *L. zhachilii* HBUAS52074T, derived from naturally fermented foods, alleviated the severity of depressive-like behaviors while enhancing intestinal barrier function and reduced inflammation in peripheral blood in CSDS mice [39]. Figure 2 summarizes the main effects of psychobiotics on peripheral inflammation in animal models of depression.

## 6. Neuroinflammation

It has been recognized for decades that neuroinflammation, particularly in cortical regions and HIPP, plays an important role in the pathophysiology of depression. Also, many studies on animal models have shown that concentrations of pro-inflammatory cytokines in these brain regions decrease in response to antidepressant treatment [84,85]. Table 4 summarizes experimental evidence on the protective effects of psychobiotics against neuroinflammation in several brain regions, in various animal models of depression.

Numerous studies have shown that psychobiotics attenuate inflammation in the brain, as evidenced by decreased levels of pro-inflammatory cytokines, mainly TNF-α, IL-1β, and IL-6, an increased expression of anti-inflammatory cytokine IL-10, reduced expression of Toll-like receptors (TLR4, TLR5), and diminished Iba1 reactivity (Table 4).

**Figure 3:**
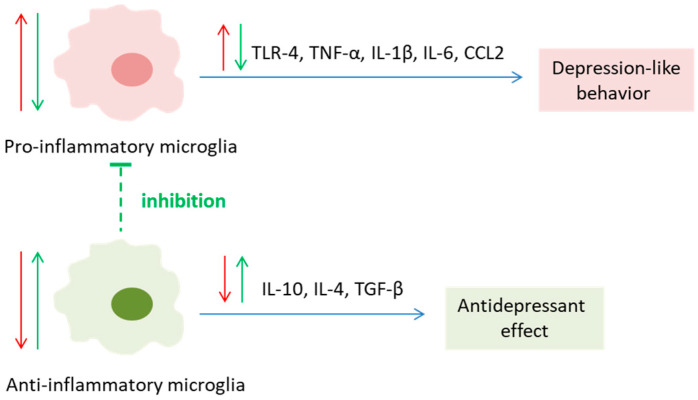
Overview of mediators of neuroinflammation affected by depression (red arrows) and psychobiotic treatment (green arrows) in the brain: TLR—Toll-like receptor; TNF-α—tumor necrosis factor-alpha; IL—interleukin; CCL2—chemokine (C-C motif) ligand 2; TGF-β—transforming growth factor-β.

Given the differences in anatomical and functional organization, modulations of inflammation-related molecules should be considered separately for each brain structure. The majority of studies have focused on the effects of psychobiotics on neuroinflammatory markers in the HIPP, a brain region strongly implicated in depression. Several psychobiotics have demonstrated potential in reducing TNF-α levels in HIPP. Strains reported to exert such effects include *Bifidobacterium longum* CECT 30763, *Pediococcus acidilactici* CCFM1344, *Lactobacillus zhachilii* HBUAS52074_T_, *Lactobacillus plantarum* GM11, *Lactiplantibacillus plantarum* JYLP-326, *Lactocaseibacillus rhamnosus* zz-1, and *Akkermansia muciniphila*, as well as a multi-strain psychobiotic formulation [20] (Table 4). Notably, *Bifidobacterium breve* UBBr-01 reduced TNF-α levels in HIPP only when administered as part of a multi-strain psychobiotic, whereas two studies reported no effects following standalone treatment with *Bifidobacterium breve* strains M-16V or CCFM1025. Considering the variability in treatment duration across studies, these findings suggest that strain-specific properties represent the primary determinants of TNF-α modulation in HIPP. In addition, two studies [19,20] reported no sex-specific effects from *Bacillus coagulans* Unique IS-2 or its combination with other psychobiotics on TNF- α modulation in HIPP. However, studies investigating the sex-specific effects of psychobiotic action are lacking, and further research in this field is needed. In addition to TNF-α, HIPP levels of other pro-inflammatory cytokines, IL-1β and IL-6, were also modulated by certain psychobiotics. Only four psychobiotics were shown to reduce both cytokines: *Lactiplantibacillus plantarum* JYLP-326, *Bifidobacterium breve* CCFM1025, *Lactocaseibacillus rhamnosus* zz-1, and *Weissella paramesenteroides* WpK4, with treatment durations of 3, 5, 4, and 1–4 weeks, respectively (Table 4). Notably, these observations further support the conclusion that psychobiotic strain characteristics, rather than treatment duration, are the primary determinants of the observed molecular outcomes. Corroborative evidence for attenuated neuroinflammation is also provided by reports of reduced microglial activation, as indicated by decreased expression of the microglial marker Iba1 [39,48,58] and the proportion of M1 and M2 microglia phenotypes [22]. Ma et al. recently reported that *L. plantarum* CR12 attenuated CUMS-induced microglial activation in the HIPP [22]. Microglial activation is reported in both clinically depressed patients and animal models. In fact, microglia, the predominant immune cells in the brain, play such an important role in depression that depression itself has been considered a microglia-associated disorder (microgliopathy) for a decade [86]. Microglia are resident brain cells that regulate inflammation, synaptic plasticity, and the formation of neural networks, all of which are implicated in depression [82]. In addition, inflammation may contribute to Trp metabolism through the kynurenine (Kyn) pathway, which is also implicated in depression (it will be discussed in more detail in Section 8. Tryptophan Metabolism). Interestingly, large amounts of quinolinic acid (QUIN), a neurotoxic Trp metabolite that has a strong excitotoxic role through enhancing NMDAR activation, are produced and secreted by activated microglia [87]. Bearing in mind the important role of microglia in depressive symptoms, it is safe to conclude that psychobiotic-driven suppression of microglial activation contributes to their antidepressant action (Figure 3).

Studies investigating changes in anti-inflammatory cytokines in the HIPP following psychobiotic treatment remain limited. *Bifidobacterium breve* CCFM1025 and *Lactiplantibacillus plantarum* CR12 were reported to increase IL-10 levels in HIPP. However, further research is required to substantiate these findings and to clarify their reproducibility and strain specificity.

Recently, it was shown that a combined probiotic containing *L. rhamnosus* HN001 and *B. animalis* subsp. *lactis* HN019 suppressed the activation of NLRP3 inflammasome in HIPP and cortex in CUMS rats [83]. Inhibition of NLRP3 inflammasome, which has been considered a key contributor to the development of neuroinflammation, has emerged as a novel therapeutic strategy for depression [88]. Interestingly, even parabiotics may exert protective effects. Thus, heat-sterilized *B. breve* M-16V suppressed depression-like behavior and neuroinflammation induced by CSDS [50]. This is very captivating considering that heat-sterilized *B. breve* M-16V might constitute a potential functional food to prevent inflammation-associated diseases, including depression. These insights are of great importance, as they unveil a novel avenue for the development of functional foods aimed at psychiatric disease prevention.

Studies investigating neuroinflammation-related effects of psychobiotics in the PFC remain relatively scarce compared with those focusing on the HIPP. One notable study examined the effects of *Lactiplantibacillus plantarum* P72 across different depression models, immobilization (IM) and transplantation of cultured fecal microbiota from patients with depression (cFM), revealing distinct molecular response patterns [26]. In the IM model, psychobiotic treatment reduced both TNF-α and IL-1β levels while increasing IL-10, whereas in the cFM model, *Lactiplantibacillus plantarum* P72 increased PFC IL-10 levels only. A reduction in TNF-α in the cFM model was observed exclusively following co-administration with *Bifidobacterium longum* P77. These findings indicate that the specificity of the depression paradigm plays a critical role in determining the molecular outcomes of psychobiotic interventions, underscoring the need for careful interpretation with respect to the underlying animal model. In addition, two studies [19,20] reported sex-specific effects of *Bacillus coagulans* Unique IS-2 on cortical TNF-α levels, which were not observed in the HIPP, highlighting the importance of further investigations into sex-dependent differences in psychobiotic actions within cortical regions, specifically the PFC.

## 7. Neurotransmission

Psychobiotics have been shown to exert their effects on neurotransmission through multiple mechanisms, including modulation of neurotransmitter levels in both central and peripheral tissues, as well as regulation of the expression of neurotransmitter receptors and the metabolism and bioavailability of neurotransmitters (Table 5).

### 7.1. Serotonin

The majority of studies examining the impact of psychobiotics on neurotransmission have investigated the serotonergic system since 5-HT has been considered a key player in depressive disorders since the late 1960s, when the serotonergic theory of depression was first communicated. Despite being increasingly doubted through the decades, as evidence has emerged for the influence of other neurotransmitters, such as dopamine, GABA, noradrenaline, and glutamate, 5-HT is still recognized as one of the key factors in the pathophysiology of depression [90]. This is further supported by the fact that selective serotonin reuptake inhibitors (SSRIs), which block the 5-HT transporter (5-HTT) and prolong the time that 5-HT activates postsynaptic 5-HT receptors, are still the most commonly prescribed antidepressants. Numerous studies have reported that psychobiotic administration elevates 5-HT levels in serum [26,33,38,39,44] and whole brain [24,27,29,34,38,39,74], as well as in brain regions highly implicated in depression: HIPP [17,25,30,32,44,47,54,58] and PFC [26,32,47] (Table 5). Regarding central 5-HT levels under psychobiotic treatment, the most consistent results have been found in the whole brain, where eight studies reported an increase in 5-HT levels, whereas only one study reported no effect. Predominantly consistent results have been reported for the HIPP as well, with eight studies reporting an increase and only two studies reporting no effect on 5-HT levels. On the other hand, results in cortical regions are conflicting, with equal numbers of studies reporting increases (four) and no effect (four). Experimental data also show that psychobiotics increase the levels of 5-hydroxytryptophan (5-HTP), the direct precursor of 5-HT, in the intestine [17,34,43], serum [17,43,55,89], cortex [43], and HIPP [17] (Table 6, Section 8). Interestingly, more than 90% of 5-HT is synthesized in the gastrointestinal tract by specialized endocrine cells called enterochromaffin cells. While peripheral 5-HT is unable to cross the blood–brain barrier (BBB), its precursor, 5-HTP, can penetrate the BBB [91] and influence 5-HT production in the brain. Research has shown that gut microbiota can directly interact with enterochromaffin cells, trigger the expression of Trp hydroxylase 1 (TPH1), which metabolizes Trp to 5-HTP, the first and rate-limiting step in the biosynthesis of 5-HT [92]. Gao et al. reported that *Lactococcus lactis* WHH2078 upregulated colonic *Tph1* gene expression and increased the colonic, serum, and HIPP levels of 5-HTP, which resulted in restoring the 5-HT level in HIPP and alleviated CUMS-induced depression in mice [17]. Concerning TPH2, the isoenzyme primarily expressed in the serotonergic neurons of the brain, Gao et al. reported that *L. lactis* WHH2078 did not affect its expression [17], while Zhu et al. have found that *L. plantarum* JYLP-326A upregulated TPH2 in HIPP of CUMS mice [25]. A highly compelling study published this year has shown that a high-yielding strain for 5-HTP production, created using genetic engineering, alleviated depressive-like behaviors in mice by increasing 5-HT levels in both the gut and brain, repairing neurological abnormalities, inhibiting inflammation, elevating SCFA concentrations, and modulating gut microbiota dysbiosis [93]. Regarding 5-HT receptors, Baek et al. reported that *L. plantarum* P72 and *Bifidobacterium longum* P77 increased the expression of 5-HT_1A_R and 5-HT_1B_R in PFC [26], while Zhu et al. showed that *L. plantarum* JYLP-326 elevated 5-HT_1A_R in HIPP [25], in parallel with attenuating depressive- and anxiety-like behaviors in animal models. Interestingly, as far as we know, there are no experimental data on the psychobiotic effect on 5-HTT, the main target of SSRIs, the most commonly prescribed antidepressants.

### 7.2. Dopamine

Dopamine (DA) release and DA metabolism are changed in response to stressful stimuli. DA release can be enhanced or inhibited on the basis of the intensity, duration, and avoidability of the stressor. Mild to moderate stressors that are novel, short-lasting, or controllable have activating effects on DA release, while intense, chronic, and unpredictable stressors have inhibitory effects on DA [94]. For example, CSDS was shown to increase DA plasma levels and reduce DA receptors, probably to attenuate excessive DA signaling under stress [51]. Administration of *B. longum* normalized plasma DA levels and reversed the CSDS-induced reduction of D1, D2L, and D2S receptor expression in the intestine. Furthermore, *B. longum* activated the intestinal host DA catabolic enzymes through restoration of catechol-O-methyltransferase (COMT) expression [51]. In addition, *B. longum* restored reduced postsynaptic dopamine D2L receptor levels in PFC and normalized the expression of the enzymes involved in DA catabolism (COMT in the striatum and MAOA in PFC), which may contribute to the normalization of DA signaling and turnover, and may be, at least partially, responsible for the psychobiotic ability of the bacterium [51]. On the other hand, several studies involving animal models based on chronic, unpredictable stress reported decreased DA levels in serum and brain of animals with depressive symptoms, as well as control levels in animals treated with psychobiotics in parallel with stress exposure [18,24,38]. Regarding the effect of psychobiotics on catabolism of DA, the results lack consistency. Huang et al. reported decreased levels of 3,4-dihydroxyphenylacetic acid (DOPAC), a product of DA breakdown, in serum and brain of CUMS rats after psychobiotic treatment [38], while Daugé et al. showed brain region-specific effects of a psychobiotic—no change was detected in HIPP, while in striatum, the psychobiotic increased DOPAC levels in MS rats [65].

### 7.3. GABA

**Figure 4:**
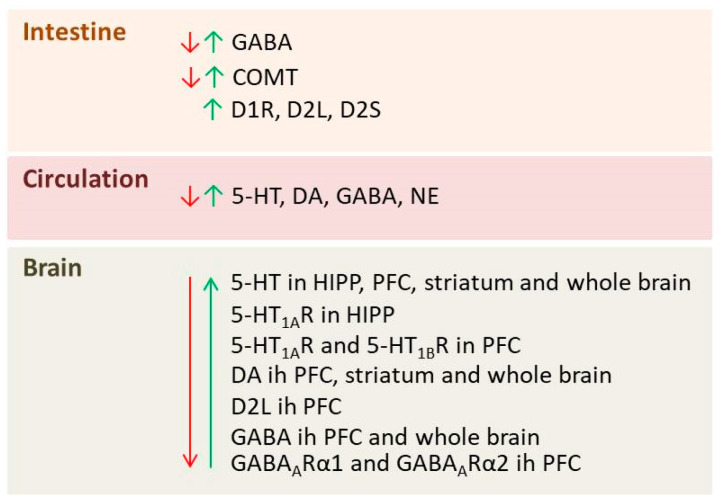
Overview of parameters related to neurotransmission affected by depression (red arrows) and psychobiotic treatment (green arrows) in the gut, circulation and brain: GABA—gamma-aminobutyric acid; COMT—catechol-O-methyltransferase; D1R—D1 dopamine receptor; D2L and D2S—two isoforms of D2 dopamine receptors; 5-HT—5-hydroxytryptamine; DA—dopamine; NE—norepinephrine; HIPP—hippocampus; PFC—prefrontal cortex; 5-HT_1A_R—serotonin 1A receptor; 5-HT_1B_R—serotonin 1B receptor; GABA_A_Rα1—gamma-aminobutyric acid type A receptor subunit alpha1; GABA_A_Rα2—gamma-aminobutyric acid type A receptor subunit alpha2.

Dysregulation of the GABAergic system, the principal inhibitory neurotransmitter system in brain circuits, is highly implicated in depression [95]. In our laboratory, with the use of comprehensive metabolomic analysis, we identified alterations in HIPP GABA levels as a hallmark of depressive-like behavior in chronically socially (long-term) isolated rats, while their restoration represented a hallmark of antidepressant treatment effectiveness [96]. These GABA alterations were also reflected by diminished expression of the calcium-binding protein parvalbumin (PV) in the medial PFC [97], as well as in the most abundant subclass of PV-immunoreactive GABAergic interneurons within the dorsal HIPP [98]. Moreover, effective treatment with different classes of antidepressants tends to restore GABA levels [99], as well as PV expression [97] and the expression of the predominant isoform of the rate-limiting GABA-synthesizing enzyme, glutamate decarboxylase 67, in the dorsal HIPP [98,100]. In addition, cerebrospinal fluid levels of GABA were found to be lower in patients with depression compared with healthy controls [101]. Hence, based on the growing body of evidence, dysregulated inhibitory GABAergic signaling has emerged as one of the key targets in recent efforts to develop novel antidepressant modalities. Considering that gut microbiota is an important regulator of GABA [102], it is reasonable to pursue microbiota-based therapeutics capable of normalizing GABA levels in depression. Our recent experiments have shown that a postbiotic of the newly isolated and characterized GABA-producing bacterial strain *Phocaeicola vulgatus* NGB218 prevented depression- and anxiety-like behavior in CUMS rats [63]. Li et al. reported that *Lactiplantibacillus plantarum* GOLDGUT-HNU082 increased GABA levels in colon and serum, but had no effect on brain GABA levels in CUMS mice [24]. However, Baek et al. recently reported that *L. plantarum* P72 and *B. longum* P77, as well as their combination, increased GABA and its receptors GABA_A_Rα1 and GABA_A_Rα2 in PFC in IM and cFM models of depression [26]. Figure 4 summarizes the main effects of psychobiotics on neurotransmission in animal models of depression.

## 8. Tryptophan Metabolism

Trp-derived metabolites play an important role in host physiology by preserving intestinal homeostasis and the fine-tuning of immune and metabolic responses. The perturbations of gut microbial Trp metabolism may affect the course of both intestinal and extraintestinal disorders, including stress-induced depression [103]. Alterations in Trp levels, both in peripheral circulation and in the CNS, represent one of the most consistently reported metabolite and amino acid changes in depression. Studies demonstrating that the administration of various psychobiotics in animal models of depression affects the concentration of different Trp metabolites in the gut, circulation, and brain are presented in Table 6.

**Table 6:** The effects of psychobiotics on Trp metabolism in animal models of depression.

Psychobiotic		Treatment Duration (Weeks)	Animal	Sex (M/F)	Animal Model	Sample	Effect of Psychobiotic on Trp Metabolism in Depression Model	Ref.
Genus	Strain							
*Lactobacillus*	*Lactiplantibacillus plantarum* KLDS	5	Mice	M	CUMS	Intestine	↑ 5-HTP; ↑ indolepyruvate; ↑ melatonin; ↑ 5-HIAA	[34]
	*Lactiplantibacillus plantarum* D-9	2	Mice	M	CUMS	Hypothalamus	↑ 5-HT/Trp ratio; ↑ 5-HIAA/5-HT ratio	[23]
						Serum	No effect: Kyn/Trp ratio	
	*Lactiplantibacillus plantarum* JYLP-326	3	Mice	M	CUMS	HIPP	↑ TPH2	[25]
	*Lacticaseibacillus rhamnosus* KY16	7	Mice	M	CUMS	Serum	↑ 5-HTP; ↑ 5-HT	[43]
						Colon	↑ 5-HTP; ↑ *Tph1* No effect: 5-HT	
						Cortex	↑ 5-HTP; ↑ 5-HT	
						HIPP	↑ *Tph2*	
	*Lactocaseibacillus rhamnosus* zz-1	4	Mice	M	CUMS	HIPP	↑ 5-HT; no effect: Kyn; Trp	[44]
						Serum	↑ 5-HT; ↓ Kyn; ↓ Kyn/Trp ratio	
	*Lactobacillus rhamnosus* HN001	6	Rats	M	CUMS	Brain	↑ 5-HT; ↓ 5-HIAA	[38]
	*Lactobacillus reuteri* ATG-F4	4	Mice	M	CUS	Serum	No effect Trp, 5-HT, 5-HIAA, Kyn, KA; ↑ 5-HTP; ↑ Tryptamine	[89]
						Brain	No effect 5-HTP, Kyn, KA; ↓ Trp; ↓ 5-HT; ↓ 5-HIAA; ↓ Tryptamine	
*Lactococcus*	*Lactococcus lactis* ZFM559	5	Mice	M	CUMS	Brainstem	↓ 5-HIAA/5-HT ration; ↑ Trp No effect: 5-HT, 5-HTP, *Tph2*	[55]
						Serum	↑ 5-HTP No effect: 5-HT	
						Colon	↑ 5-HT No effect: 5-HTP, *Tph1*	
	*Lactococcus lactis* WHH2078	5	Mice	M	CUMS	HIPP	↑ 5-HT; ↑ 5-HTP; no effect TPH2	[17]
						Serum	↑ 5-HTP	
						Colon	↑ 5-HTP; ↑ TPH1	
*Bacillus*	*Bacillus licheniformis*	4	Rats	M	CUMS	Whole brain	↓ Kyn; no effect: 5-HT	[60]
	*Bacillus coagulans* Unique IS-2	6	Rats	M	MS and CUMS	Plasma	No effect Trp; ↓ Kyn; ↑ KA; ↓ 3-hydroxyanthranilic acid	[19]
				F			No effect Trp, KA; ↓ Kyn; ↓ 3-hydroxyanthranilic acid	
*Bifidobacterium*	*Bifidobacterium lactis* HN019	6	Rats	M	CUMS	Brain	↑ 5-HT; ↓ 5-HIAA	[38]
*Lactobacillus*, *Bifidobacterium*	HN001 and HN019	6	Rats	M	CUMS	Brain	↑ 5-HT; ↓ 5-HIAA	[38]
*Roseburia*, *Bacteroides*, *Eubacterium*	Multi-strain probiotic: *Roseburia inulinivorans*, *Bacteroides uniformis*, and *Eubacterium rectale*	4	Rats	M	CUMS	Brain	↑ Kyn; No effect: Trp	[67]
*Lactobacillus*, *Bifidobacterium*, *Bacillus*	Multi-strain probiotic: *B. coagulans Unique* IS-2, *L. plantarum* UBLP-40, *L. rhamnosus* UBLR-58, *B. lactis* UBBLa-70, *B. breve* UBBr-01, *B. infantis* UBBI-01	6	Rats	M	MS and CUMS	Plasma	No effect Trp; ↓ Kyn; ↑ KA; ↓ 3-hydroxyanthranilic acid	[20]
				F			No effect Trp; ↓ Kyn; ↓ KA ↓ 3-hydroxyanthranilic acid	

This table summarizes the results of studies which examined the effects of psychobiotics on Trp metabolism in animal models of depression. Studies investigating psychobiotics from the same genus are grouped together. The genera are arranged by the number of studies in which they have been investigated, with those most frequently used as psychobiotics listed first. The columns specify the genus of the psychobiotic, the strain used, animal subject in depression modeling (mice/rats), the sex of the animals (male (M) or female (F)), the procedure employed to induce depression (CUMS, MS, CUS), the sample used for Trp-metabolism parameter measurement (brainstem, colon, serum, etc.) and the molecular changes observed after psychobiotic treatment, with ↑ denoting increases and ↓ denoting decreases relative to the depression animal model: CUMS—chronic unpredictable mild stress; CUS—chronic unpredictable stress; MS—maternal separation; HIPP—hippocampus; 5-HTP—5-hydroxytryptophan; 5-HIAA—5-hydroxyindoleacetic acid; 5-HT—5-hydroxytryptamine; Trp—tryptophan; Kyn—kynurenine; TPH2—tryptophan hydroxylase 2; TPH1—tryptophan hydroxylase 1; KA—kynurenic acid.

**Figure 5:**
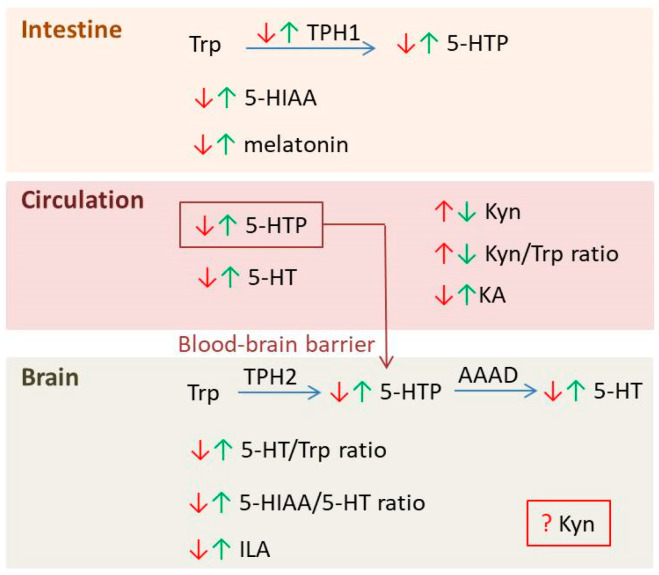
Overview of TRP metabolites affected by depression (red arrows) and psychobiotic treatment (green arrows) in the gut, circulation and brain: Trp—tryptophan; TPH1—tryptophan hydroxylase 1; 5-HTP—5-hydroxytryptophan; 5-HIAA—5-hydroxyindoleacetic acid; 5-HT—5-hydroxytryptamine; Kyn—kynurenine; KA—kynurenic acid; TPH2—tryptophan hydroxylase 2; AAAD—aromatic L-amino acid decarboxylase; ILA—indole-3-lactic acid.

Indole-3-lactic acid (ILA) is undoubtedly a compound of interest when considering Trp metabolite-mediated protective effects of psychobiotics. A recent study revealed a significant reduction in ILA levels in HIPP of depressed mice, which was ameliorated by the psychobiotic *B. breve*. Importantly, the antidepressant effects were nullified in the mutants of *B. breve* missing lactate dehydrogenase, the enzyme responsible for ILA production [46]. A recent pharmacokinetic study revealed that ILA can be efficiently absorbed from the gastrointestinal tract, cross the BBB, and maintain a relatively stable concentration in the brain, where it plays a role in regulating brain immune homeostasis [104]. In addition to the indole pathway, Trp is processed through two other metabolic routes relevant to depression: the 5-HT and Kyn pathways. Its conversion into 5-HT supports mood regulation, while diversion into the Kyn pathway can lead to neuroactive metabolites associated with neuroprotection (kynurenic acid, KYNA) and neurotoxicity (quinolinic acid, QUIN). An imbalance between these pathways, often influenced by stress, immune activation, or gut microbiota, has been implicated in the development and progression of depressive disorders. Maintaining a healthy Trp metabolic balance is therefore crucial for emotional and neurological well-being [105]. Numerous studies indicate abnormalities in brain Kyn levels in depression (Table 6), although it remains controversial whether these levels are increased or decreased. Regarding plasma/serum levels of Kyn, results have been fairly consistent, with most studies reporting decreased levels, although Lee et al. reported no effect of a probiotic [89]. The situation is more complex in the brain, where conflicting results have been noted. Feng et al. reported a decrease in brain Kyn levels following *Bacillus licheniformis* administration [60], whereas Meng et al. observed an increase after treatment with a multi-strain probiotic [67]. Differences in experimental design should be taken into account when discussing these conflicting findings. Feng et al. administered a probiotic in parallel with CUMS exposure (an 8-week CUMS protocol with probiotic treatment during the last 4 weeks), while Meng et al. administered a probiotic after CUMS exposure (a 4-week CUMS protocol followed by 4 weeks of treatment). It should also be noted that Feng et al. reported increased brain Kyn levels in CUMS rats, whereas Meng et al. found no differences in Kyn levels between CUMS and control rats. It is possible that the 4-week recovery period after CUMS was sufficient to normalize Kyn levels, or the 4-week CUMS protocol was not long enough to induce changes in Kyn levels in the brain. Based on the results published to date, it can be concluded that psychobiotics have the potential to modulate the Kyn metabolic pathway; however, the role and regulation of Kyn in the brain in depression, as well as under psychobiotic treatment, require further investigation. Figure 5 summarizes the main effects of psychobiotics on Trp metabolism in animal models of depression.

## 9. Linking Preclinical Findings with Human Data

Accumulating preclinical findings presented here provide evidence for the protective effects of psychobiotics against behavioral and molecular changes in various animal models of depression. Some of those findings have been corroborated in human studies; however, the pathways to translation from preclinical to clinical use are not always straightforward. A recent systematic review of randomized clinical trials reported that psychobiotics, particularly strains of *Lactobacillus* and *Bifidobacterium*, can offer significant benefits in managing psychiatric disorders [106]. Numerous studies have shown significantly reduced blood or salivary cortisol levels in human participants after consuming different *Lactobacillus* or *Bifidobacterium* strains [107,108,109,110,111,112], which is in line with the beneficial effects of psychobiotics on the HPA axis presented in Section 4. Also, several clinical studies demonstrated an anti-inflammatory effect of psychobiotics. Chong et al. have shown in a randomized, double-blind, placebo-controlled study that *Lactobacillus plantarum* DR7 alleviated stress and anxiety in adults by reducing plasma levels of cortisol and pro-inflammatory cytokines IFN-γ and TNF-α. The administration of DR7 downregulated the Kyn pathway and upregulated the 5-HT pathway, judging by decreased blood levels of indoleamine 2,3-dioxygenase and tryptophan 2,3-dioxygenase, and by increased levels of the TPH enzyme, respectively [112]. Decreased plasma levels of IFN-γ and TNF-α were also detected in stressed adults after treatment with *Lactobacillus plantarum* P8 [113]. Lee et al. found that a probiotic containing *Lactobacillus reuteri* NK33 and *Bifidobacterium adolescentis* NK98 lowered IL-6 levels in serum and alleviated depression and anxiety in adults with psychological stress and subclinical symptoms of depression [114]. Rudzki et al. reported that *Lactobacillus plantarum* 299v decreased Kyn plasma concentrations and improved cognitive functions in patients with major depression [115]. However, it should be noted that a considerable number of studies, estimated by some to account for up to one-third [116], have failed to demonstrate significant clinical benefits from psychobiotic consumption. The high heterogeneity of bacterial strains used in the studies definitely contributes to discrepancies within results and hinders the ability to draw meaningful inferences. The placebo effect, which has been demonstrated in clinical studies on probiotics [117], further complicates drawing general conclusions on the usefulness of psychobiotics in patients suffering from depression. When discussing the translational value of preclinical results, it is important to point out the differences in gut microbiota composition between rodents used as subjects for modeling depression in preclinical studies and humans, as well as individual differences in microbiome composition. Furthermore, the challenges and constraints associated with probiotic use in general should always be taken into account. Strain-specific efficacy and variability in human responses, influenced by genetics, diet, and gut microbiota composition, should be considered [118]. Taking all this into account, caution should be exercised when discussing preclinical findings and considering their clinical translation. To make progress towards the clinical use of psychobiotics, a deeper mechanistic insight into host–microbe interactions within the MGBA axis is required, as well as an understanding of how host genetic variants and environmental factors influence therapeutic outcomes [119].

## 10. Conclusions

**Figure 6:**
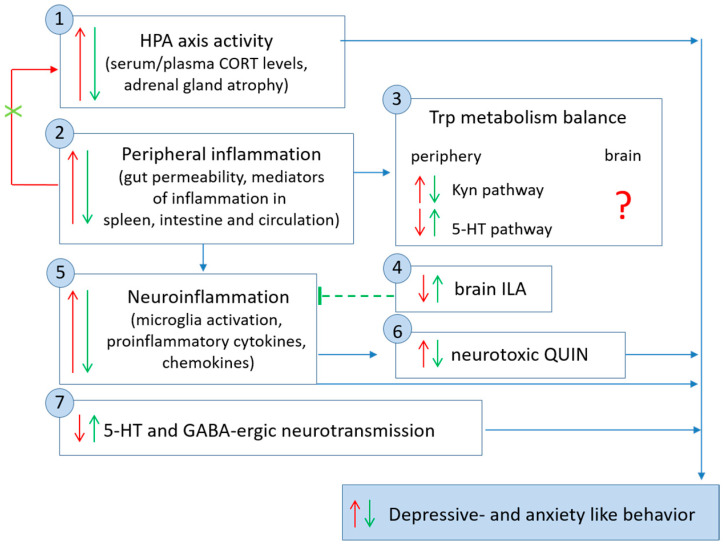
Summary of molecular changes associated with depressive symptomatology (red arrows) and protective effects of psychobiotics (green arrows). Growing evidence suggests that protective effects of psychobiotics against depressive- and anxiety-like behaviors in animal models of depression are mediated through protective changes in molecular levels: (1) the HPA axis—psychobiotics have been shown to mitigate HPA axis hyperactivity associated with depressive-like symptoms in animal models; (2) peripheral inflammation—psychobiotics have been shown to enhance intestinal barrier integrity and decrease levels of pro-inflammatory mediators in the spleen, intestine and circulation; peripheral inflammation, particularly in the gut, can activate the HPA axis; psychobiotics may attenuate HPA activity by preventing gut leakiness and a consequent increase in circulating LPS levels; (3) Trp metabolism balance—inflammation may contribute to Trp metabolism through the Kyn pathway, downregulating the 5-HT pathway; the anti-inflammatory effects of psychobiotics affect Trp metabolic balance by downregulating Kyn and upregulating the 5-HT pathway; (4) psychobiotics have been shown to ameliorate decreases in brain levels of ILA, which plays a role in regulation of immune homeostasis in the brain; (5) neuroinflammation—psychobiotics have been shown to attenuate microglial activation and decrease levels of pro-inflammatory cytokines and chemokines in the brain; (6) psychobiotics have been reported to protect against QUIN, a neurotoxic Trp metabolite produced by activated microglia, by attenuating its activation; (7) neurotransmission—psychobiotics have been shown to exert protective effects against dysregulated 5-HT and GABA-ergic neurotransmission. HPA—hypothalamic-pituitary-adrenal axis; CORT—corticosterone; Kyn—kynurenine; Trp—tryptophan; 5-HT—5-hydroxytryptamine; ILA—indole-3-lactic acid; QUIN—quinolinic acid; GABA—gamma-aminobutyric acid.

This review summarizes experimental evidence that psychobiotics alleviate molecular changes related to the HPA axis, peripheral inflammation, neuroinflammation, neurotransmission, and Trp metabolism, while suppressing depressive- and anxiety-like behavior in animal models of depression (Figure 6). Psychobiotics have been repeatedly reported to normalize levels of CRH, ACTH, and CORT; decrease levels of mediators of inflammation in the intestine, circulation, and brain; normalize the levels of neurotransmitters and their receptors; and regulate Trp metabolism in various animal models of depression.

Taking these protective effects into account, it is evident that probiotics represent a fertile ground for the development of supportive therapies targeting depressive disorders. The main challenges in this field include the considerable diversity of studied probiotic strains, which are often insufficiently characterized, as well as the lack of mechanistic studies that would identify specific bacterial metabolites responsible for the antidepressant effect. Although certain metabolites, including neurotransmitters (5-HT, GABA, DA) and SCFAs, have been recognized as potentially responsible for this effect and are under the loop of recent studies, unequivocal experimental evidence is still lacking. However, despite these issues, results summarized in this review demonstrate that growing scientific interest in exploiting probiotics as a potential reservoir for the development of supportive therapies targeting depressive disorders is well founded.

## 11. Future Directions

To definitely settle the questions surrounding the therapeutic benefits of microbiota-targeted therapies for depression, well-designed, large-scale randomized controlled trials are needed. Future research should prioritize reducing heterogeneity in psychobiotic strain selection and expanding the range of molecular markers assessed. In line with this, multi-omics techniques are powerful tools that could substantially contribute to comprehensive characterization of psychobiotic-derived metabolites and the identification of those associated with antidepressant effects. In addition, systematic evaluation of treatment duration effects will be critical for enabling meaningful comparisons across studies and for elucidating the principal biochemical mechanisms by which psychobiotics contribute to depression treatment. Moreover, future studies should place greater emphasis on defining the concentrations of psychobiotic-derived active metabolites suspected to exert an antidepressant effect, as well as on elucidating the temporal dynamics of molecular changes under psychobiotic exposure. A future trend with promising opportunities is developing psychobiotics that target multiple aspects of depression pathogenesis using genetic engineering. Bioengineered psychobiotics will pave the way for effective therapeutic solutions in the form of next-generation biotherapeutics and hold great potential for a major breakthrough in the rapid advancement of microbiota-based therapies.

## Figures and Tables

**Table 1:** The effects of psychobiotics on depression- and anxiety-like behaviors in animal models of depression.

Psychobiotic		Treatment Duration (Weeks)	Animal	Sex (M/F)	Animal Model	Antidepressant Effect	Anxiolytic Effect	Ref.
Genus	Strain							
*Lactobacillus*	*Lactiplantibacillus plantarum* CR12	3	Mice	F	CUMS	TST, SPT, FST		[22]
	*Lactiplantibacillus plantarum* D-9	2	Mice	M	CUMS	TST, SPT, FST	OFT, EPM	[23]
	*Lactiplantibacillus plantarum* GOLDGUT-HNU082	8	Mice	M	CUMS	TST, SPT, FST	OFT	[24]
	*Lactiplantibacillus plantarum* JYLP-326	3	Mice	M	CUMS	TST, SPT	OFT	[25]
	*Lactiplantibacillus plantarum* P72	1	Mice	M	IM	TST	OFT, EPM	[26]
	*Lactiplantibacillus plantarum* P72				cFM	TST	OFT, EPM	
	*Lactiplantibacillus plantarum* R6-3	8	Mice	M	CUMS	TST, SPT, FST		[27]
	*Lactiplantibacillus plantarum* WH021	4	Mice	M	LPS-D	SPT	OFT	[28]
	*Lactobacillus fermentum* PS150	4	Rats	M	CUS	FST	EPM	[29]
	*Lactobacillus helveticus* NS8	3.6 (25 days)	Rats	M	CRS	SPT	OFT, EPM	[30]
	*Lactobacillus paracasei* Lpc-37	5	Mice	M	CRS	FST		[31]
	*Lactobacillus plantarum* LP12407						OFT	
	*Lactobacillus paracasei* PS23	6	Mice	M	CORT-D	SPT, FST	OFT	[32]
	*Lactobacillus plantarum* GM11	3	Rats	M	CUMS	SPT, FST		[33]
	*Lactobacillus plantarum* KLDS1.0386	5	Mice	M	CUMS	TST, SPT, FST	OFT	[34]
	*Lactobacillus plantarum* MTCC 9510	4	Mice	M	CUMS and SD	TST, FST	EZM	[35]
	*Lactobacillus plantarum* PS128	4	Mice	M	MS	SPT, FST	OFT	[18]
	*Lactobacillus plantarum* WLPL04	4	Mice	M	CRS	FST	OFT, EPM	[36]
	*Lactobacillus rhamnosus* GG (ATCC 53103)	2	Rats	M	CUMS	FST		[37]
	*Lactobacillus rhamnosus* HN001	6	Rats	M	CUMS		OFT, EPM	[38]
	*Lactobacillus zhachilii* HBUAS52074T	4	Mice	M	CSDS	TST, SPT, SIT	OFT	[39]
	*Lacticaseibacillus casei* IDCC 3451	10	Mice	M	CUMS	TST, SPT, FST	OFT, EZM	[40]
	*Lacticaseibacillus rhamnosus* IDCC3201	9	Mice	M	CUMS		EPM	[41]
	*Lacticaseibacillus rhamnosus* JB-1	8	Rats	M	CUMS		EPM	[42]
	*Lacticaseibacillus rhamnosus* KY16	7	Mice	M	CUMS	SPT, FST	OFT, EPM	[43]
	*Lactocaseibacillus rhamnosus* zz-1	4	Mice	M	CUMS	TST, SPT	EPM	[44]
*Bifidobacterium*	*Bifidobacterium adolescentis* NGB329	8	Rats	M	CUMS	SPT	EPM, MBT	[45]
	*Bifidobacterium breve* Bre1025	4	Mice	M	CUMS	TST, FST	OFT	[46]
	*Bifidobacterium breve* CCFM1025	5	Mice	M	CUMS	TST, SPT, FST	OFT, EPM	[47]
	*Bifidobacterium breve* CCFM1025	5	Mice	M	CUMS	FST	OFT, EPM, LDB	[48]
	*Bifidobacterium breve* CCFM1025	6	Mice	M	CUMS	TST, FST	OFT, EPM	[49]
	*Bifidobacterium breve* M-16V	4.7 (33 days)	Mice	M	CSDS	SIT		[50]
	*Bifidobacterium infantis*	7	Rats	M/F	MS	M–/ F-FST		[21]
	*Bifidobacterium lactis* HN019	6	Rats	M	CUMS		OFT, EPM	[38]
	*Bifidobacterium longum* CECT 30763	6.6 (46 days)	Mice	M	CSDS	SPT, FST	LDB	[51]
	*Bifidobacterium longum* P77	1	Mice	M	IM	TST	OFT, EPM	[26]
	*Bifidobacterium longum* P77				cFM	TST	OFT, EPM	[26]
	*Bifidobacterium pseudocatenulatum* CECT 7765	3	Mice	M	MS		EPM	[52]
*Lactococcus*	*Lactococcus lactis* subsp. *cremoris* LL95	1	Mice	M	LPS-D	FST		[53]
	*Lactococcus lactis WHH2078*	5	Mice	M	CUMS	TST, SPT, FST	OFT	[17]
	*Lactococcus lactis* WHH2078	4	Mice	M	CRS	TST, SPT, FST	OFT	[54]
	*Lactococcus lactis* ZFM559	5	Mice	M	CUMS	TST, SPT	OFT	[55]
*Faecalibacterium*	*Faecalibacterium prausnitzii* ATCC 27766	6	Rats	M	CUMS+ ACTH	SPT, FST		[56]
	*Faecalibacterium prausnitzii* ATCC 27766	4	Rats	M	CUMS	FST	OFT, EPM	[57]
*Akkermansia*	*Akkermansia muciniphila*	every 3 days for 2 weeks	Mice	F	mALPS	TST, FST	OFT	[58]
	*Akkermansia muciniphila*	3	Mice	M	CRS	TST, FST	OFT	[59]
*Bacillus*	*Bacillus coagulans* Unique IS-2	6	Rats	M/F	MS and CUMS	SPT, FST	EPM	[19]
	*Bacillus licheniformis*	4	Rats	M	CUMS	FST	EPM	[60]
*Pediococcus*	*Pediococcus acidilactici* CCFM1344	4	Mice	No info.	CUMS	TST	OFT, EPM	[61]
*Weissella*	*Weissella paramesenteroides* WpK4	1.4 (10 days)	Mice	F	CRS	TST	EPM	[62]
*Phocaeicola*	*Phocaeicola vulgatus* NGB218	8	Rats	M	CUMS	SPT	EPM, MBT	[63]
*Lactobacillus*, *Bifidobacterium*	*Lactobacillus rhamnosus* HN001 and *Bifidobacterium lactis* HN019	6	Rats	M	CUMS		OFT, EPM	[38]
*Lactobacillus*, *Streptococcus*	*Lactobacillus plantarum* NBIMCC 8767 + *Streptococcus thermophilus* NBIMCC 8258	2	Rats	M	CUMS	SPT, FST		[64]
*Bacillus*, *Lactobacillus*, *Bifidobacterium*	Multi-strain probiotic formulation: *Bacillus coagulans* Unique IS-2, *Lactiplantibacillus plantarum* UBLP-40, *Lactobacillus rhamnosus* UBLR-58, *Bifidobacterium lactis* UBBLa-70, *Bifidobacterium breve* UBBr-01, *Bifidobacterium infantis* UBBI-01	6	Rats	M/F	MS and CUMS	SPT, FST	EPM	[20]
*Lactobacillus*, *Bifidobacterium*, *Lactococcus*, *Streptococcus*	Multi-strain probiotic formulation: *Lactobacillus helveticus* LA 102, *Bifidobacterium longum* LA 101, *Lactococcus lactis* LA 103, *Streptococcus thermophilus* LA 104	5	Rats	M	MS	FST	OFT, LDB	[65]
*Lactobacillus*, *Bifidobacterium*	Multi-strain probiotic formulation: *Lactobacillus plantarum* ATCC 793, *Bifidobacterium longum* ATCC 15707	11	Mice	M	CUS	FST	OFT	[66]
*Lactobacillus*, *Bifidobacterium*	P72+P77	1	Mice	M	IM	TST	OFT, EPM	[26]
					cFM	TST	OFT, EPM	
*Roseburia*, *Bacteroides*, *Eubacterium*	Multi-strain probiotic: *Roseburia inulinivorans*, *Bacteroides uniformis*, and *Eubacterium rectale*	4	Rats	M	CUMS	FST	OFT, EPM	[67]

This table summarizes the results of studies examining the effects of psychobiotics on depression- and anxiety-like behaviors in animal models of depression. Studies investigating psychobiotics from the same genus are grouped together. The genera are arranged by the number of studies in which they have been investigated, with those most frequently used as psychobiotics listed first. The columns specify the genus of the psychobiotic, the strain used, the animal subject in depression modeling (mice/rats), the sex of the animals (male (M) or female (F)), and the procedure employed to induce depression (e.g., CUMS, IM, cFM). The columns “antidepressant effects” and “anxiolytic effects” highlight the behavioral tests in which the psychobiotic demonstrated antidepressant/anxiolytic effects in animal models of depression: CUMS—chronic unpredictable mild stress; IM—immobilization stress; cFM—cultured fecal microbiota of patients with depression (cFM)-induced depression; LPS-D—lipopolysaccharide-induced depression; CUS—chronic unpredictable stress; CRS—chronic restraint stress; CORT-D—corticosterone-induced depression; SD—sleep deprivation; MS—maternal separation; CSDS—chronic social defeat stress; ACTH—adrenocorticotropic hormone;; mALPS—murine alcohol-lipopolysaccharide model; TST—tail suspension test; SPT—sucrose preference test; FST—forced swim test; SIT—social interaction test; OFT—open field test; EPM—elevated plus maze; EZM—elevated zero maze; MBT—marble burying test; LDB—light-dark box.

**Table 2:** The effects of psychobiotics on HPA axis parameters in animal models of depression.

Psychobiotic		Treatment Duration (Weeks)	Animal	Sex (F/M)	Animal Model	Effect of Psychobiotic on the HPA Axis Parameters in Depression Model	Ref.
Genus	Strain						
*Lactobacillus*	*Lactobacillus helveticus* NS8	3.6 (25 days)	Rats	M	CRS	↓ plasma CORT; ↓ plasma ACTH	[30]
	*Lactobacillus plantarum* MTCC 9510	4	Mice	M	CUMS and SD	CUMS ↓ serum CORT	[35]
						SD ↓ serum CORT	
	*Lactiplantibacillus plantarum* D-9	2	Mice	M	CUMS	↓ hypothalamic CRH; ↓ serum CORT	[23]
	*Lactiplantibacillus plantarum* KLDS 1.0386	5	Mice	M	CUMS	↓ serum CORT; ↓ serum CRH	[34]
	*Lactiplantibacillus plantarum* R6-3	8	Mice	M	CUMS	↓ serum CORT	[27]
	*Lactiplantibacillus plantarum* WH021	4	Mice	M	LPS-D	↓ serum CORT	[28]
	*Lactobacillus plantarum* GM11	3	Rats	M	CUMS	↓ serum CORT; ↓ hippocampal CORT	[33]
	*Lactobacillus plantarum* PS128	4	Mice	M	MS	↓ serum CORT level	[18]
	*Lactiplantibacillus plantarum* P72	1	Mice	M	IM	↓ CORT in prefrontal cortex	[26]
	*Lactiplantibacillus plantarum* P72				cFM		
	*Lactobacillus plantarum* LP12407	5	Mice	M	CRS	No effect: plasma CORT and ACTH level	[31]
	*Lactobacillus plantarum* LP12418					No effect: plasma CORT; ↑ plasma ACTH level	
	*Lactobacillus paracasei* Lpc-37					No effect: plasma CORT and ACTH level	
	*Lacticaseibacillus rhamnosus* KY16	7	Mice	M	CUMS	↓ serum CORT	[43]
	*Lacticaseibacillus rhamnosus* TF318	4	Rats	M	CORT-D	↓ brain ACTH; ↓ brain CRH; ↓ serum CORT	[74]
	*Lactobacillus rhamnosus* zz-1	6	Mice	M	CUMS	↓ serum CRH; ↓ serum ACTH; ↓ serum CORT	[75]
	*Lactobacillus fermentum* ATCC 9338	4	Mice	M	CUMS	↓ expression of GR in the cortex, hippocampus, and medulla	[76]
	*Lactobacillus fermentum* PS150	4	Rats	M	CUS	↓ plasma CORT; ↓ brain CORT	[29]
	*Lactobacillus zhachilii* HBUAS52074^T^	4	Mice	M	CSDS	↓ serum CORT	[39]
*Bifidobacterium*	*Bifidobacterium breve* CCFM1025	5	Mice	M	CUMS	↓ serum CORT	[48]
	*Bifidobacterium breve* CCFM1025	5	Mice	M	CUMS	↓ hypothalamic CRH; ↓ serum CORT;	[47]
						↑ hippocampal Nr3c1	
	*Bifidobacterium longum* CCFM687	6	Mice	No info.	CUMS	↓ hypothalamic CRH; ↓ serum ACTH; ↓ serum CORT	[77]
	*Bifidobacterium longum* CECT 30763	6.6 (46 days)	Mice	M	CSDS	No effect: plasma CORT; ↓ adrenal atrophy	[51]
	*Bifidobacterium longum* P77	1	Mice	M	IM	↓ CORT in prefrontal cortex	[26]
	*Bifidobacterium longum* P77				cFM		
*Lactococcus*	*Lactococcus lactis* WHH2078	4	Mice	M	CRS	↓ plasma CORT	[54]
	*Lactococcus lactis* WHH2078	5	Mice	M	CUMS	↓ serum CORT	[17]
	*Lactococcus lactis* ZFM559	5	Mice	M	CUMS	↓ serum ACTH No effect: serum CORT, hypothalamic CRH	[55]
*Akkermansia*	*Akkermansia muciniphila*	3	Mice	M	CRS	↓ plasma CORT	[59]
*Faecalibacterium*	*Faecalibacterium prausnitzii* ATCC 27766	4	Rats	M	CUMS	↓ plasma CORT	[57]
*Mycobacterium*	*Mycobacterium vaccae*	2	Rats	M	Inescapable tail shock	No effect on serum CORT level	[78]
*Lactobacillus*, *Bifidobacterium*	P72+P77	1	Mice	M	IM	↓ CORT in prefrontal cortex	[26]
	P72+P77				cFM		
*Roseburia*, *Bacteroides*, *Eubacterium*	Multi-strain probiotic: *Roseburia inulinivorans*, *Bacteroides uniformis*, and *Eubacterium rectale*	4	Rats	M	CUMS	↓ serum CORT	[67]

This table summarizes the results of studies examining the effects of psychobiotics on the HPA axis in animal models of depression. Studies investigating psychobiotics from the same genus are grouped together. The genera are arranged by the number of studies in which they have been investigated, with those most frequently used as psychobiotics listed first. The columns specify the genus of the psychobiotic, the strain used, the animal subject in depression modeling (mice/rats), the sex of the animals (male (M) or female (F)), the procedure employed to induce depression (e.g., CSDS, LPS-D, CSDS), and the molecular changes observed after psychobiotic treatment, with ↑ denoting increases and ↓ denoting decreases relative to the depression animal model: CRS—chronic restraint stress; CUMS—chronic unpredictable mild stress; SD—sleep deprivation; LPS-D—lipopolysaccharide-induced depression; MS—maternal separation; IM—immobilization; cFM—cultured fecal microbiota of patients with depression (cFM)-induced depression; CORT-D—corticosterone-induced depression; CUS—chronic unpredictable stress; CSDS—chronic social defeat stress; ACTH—adrenocorticotropic hormone; CRH—corticotropin-releasing hormone; GR—glucocorticoid receptor; Nr3c1—nuclear receptor subfamily 3 group C member 1.

**Table 3:** The effects of psychobiotics on peripheral inflammation in animal models of depression.

Psychobiotic		Treatment Duration (Weeks)	Animal	Sex (F/M)	Animal Model	Sample	Effect of Psychobiotic on Inflammation in Depression Model	Ref.
Genus	Strain							
*Lactobacillus*	*Lactobacillus helveticus* NS8	3.6 (25 days)	Rats	M	CRS	Plasma	↑ IL-10; no effect: INF-γ; TNF-α	[30]
	*Lactobacillus plantarum* PS128	4	Mice	M	MS	Serum	↓ IL-6; ↑ IL-10 No effect: TNF-α	[18]
	*Lactiplantibacillus plantarum* D-9	2	Mice	M	CUMS	Serum	↓ IL-6; ↑ IL-10; no effect: INF-γ	[23]
	*Lactobacillus plantarum* MTCC 9510	4	Mice	M	CUMS/SD	Serum	CUMS: ↓ TNF-α SD: ↓ TNF-α	[35]
	*Lactobacillus plantarum* GM11	3	Rats	M	CUMS	Serum	No effect: IL-1β; TNF-α	[33]
	*Lactiplantibacillus plantarum* GOLDGUT-HNU082	8	Mice	M	CUMS	Serum	↓ LPS; No effect: TNF-α; IL-1β; IL-6	[24]
						Colon	↓ TNF-α; ↓ IL-6; No effect: IL-1β; LPS	
	*Lactiplantibacillus plantarum* JYLP-326	3	Mice	M	CUMS	Colon	↓ TNF-α; ↓ IL-1β; ↓ IL-6 ↓ TLR4; ↓ MyD88; ↓ p-p65/p65	[25]
	*Lactiplantibacillus plantarum* P72	1	Mice	M	IM	Colon	↓ TNF-α; ↓ IL-1β; ↓ IL-6	[26]
	*Lactiplantibacillus plantarum* P72				cFM	Colon	↓ TNF-α; ↓ IL-1β; ↓ IL-6; ↑ IL-10	
	*Lactiplantibacillus plantarum* R6-3	8	Mice	M	CUMS	Serum	↓ IL-1β; ↓ IL-6; ↑ IL-10 No effect: TNF-α	[27]
	*Lactiplantibacillus plantarum* WH021	4	Mice	M	LPS-D	Serum	↓ IL-1β; ↑ IL-10	[28]
						Colon	↓ TNF-α; ↓ IL-1β	
	*Lactobacillus fermentum* PS150	4	Rats	M	CUS	Colon	↓ INF-γ; no effect: TNF-α	[29]
	*Lacticaseibacillus rhamnosus* KY16	7	Mice	M	CUMS	Serum	↓ TNF-α; ↑ IL-10	[43]
	*Lactobacillus rhamnosus* HN001	6	Rats	M	CUMS	Serum	↓ TNF-α; ↓ IL-6	[38]
						Colon	↓ TNF-α; ↓ IL-1β; ↓ IL-18; ↓ IL-6	
	*Lactocaseibacillus rhamnosus* zz-1	4	Mice	M	CUMS	Serum	↓ LPS	[44]
	*Lactobacillus zhachilii* HBUAS52074^T^	4	Mice	M	CSDS	Serum	↓ LBP; ↓ TNF-α; ↓ IL-6; ↓INF-γ No effect: TGF-β1; IL-10	[39]
*Bifidobacterium*	*Bifidobacterium breve* CCFM1025	5	Mice	M	CUMS	Serum	↓ TNF-α	[47]
	*Bifidobacterium lactis* HN019	6	Rats	M	CUMS	Serum	↓ TNF-α; ↓ IL-6	[83]
						Colon	↓ TNF-α; ↓ IL-1β; ↓ IL-18; ↓ IL-6	
	*Bifidobacterium longum* CECT 30763	6.6 (46 days)	Mice	M	CSDS	Plasma	↓ CXCL9; ↓ CXCL10; ↓ CCL2; ↓TNF-α; ↓ IL-6	[51]
						Spleen	↓ CD4 T cells; ↑ Treg cells; ↓ M1 cells	
						Intestine	↑ Treg cells; ↓ TLR4 No effect: TLR2 and TLR5	
	*Bifidobacterium longum* P77	1	Mice	M	IM	Colon	↓ TNF-α; ↓ IL-1β; ↓ IL-6; ↑ IL-10	[26]
	*Bifidobacterium longum* P77				cFM		↓ TNF-α; ↓ IL-1β; ↓ IL-6	
	*Bifidobacterium pseudocatenulatum* CECT 7765	3	Rats	M	MS	Small intestine	↓ INF-γ No effect: TNF-α, IL-10, IL-18	[52]
*Lactococcus*	*Lactococcus lactis* ZFM559	5	Mice	M	CUMS	Serum	↑ IL-10; no effect: TNF-α	[55]
						Colon	↓ TNF-α; ↓ IL-1β; ↓ IL-6; ↑ IL-10	
*Pediococcus*	*Pediococcus acidilactici* CCFM1344	4	Mice	No info.	CUMS	Serum	↓ LPS	[61]
*Akkermansia*	*Akkermansia muciniphila*	every 3 days for 2 weeks	Mice	F	mALPS	Serum	↓ LPS; ↓TNF-α; ↓ IL-6	[58]
*Faecalibacterium*	*Faecalibacterium prausnitzii* ATCC 27766	4	Rats	M	CUMS	Plasma	↓ CRP; ↓ IL-6; ↑ IL-10	[57]
*Lactobacillus*, *Bifidobacterium*	HN001 and HN019	6	Rats	M	CUMS	Serum	↓ TNF-α; ↓ IL-6	[38]
						Colon	↓ TNF-α; ↓ IL-1β; ↓ IL-18; ↓ IL-6	
*Lactobacillus*, *Bifidobacterium*, *Lactococcus*, *Streptococcus*	*L. helveticus* LA 102, *Bifidobacterium longum* LA 101, *Lactococcus lactis* LA 103, and *Streptococcus thermophilus* LA 104	5	Rats	M	MS	Colon	No effect: TNF-α; IL-10	[65]
						Ileum	No effect: TNF-α; INF-γ; IL-10	
*Lactobacillus*, *Bifidobacterium*	*Lactobacillus rhamnosus* HN001 and *Bifidobacterium animalis* subsp. *lactis* HN019	6	Rats	M	CUMS	Colon	↓ expression of NLRP3	[83]
*Lactobacillus*, *Bifidobacterium*	P72+P77	1	Mice	M	IM	Colon	↓ TNF-α; ↓ IL-1β; ↓ IL-6	[26]
	P72+P77				cFM		↓ TNF-α; ↓ IL-1β; ↓ IL-6	
*Roseburia*, *Bacteroides*, *Subacterium*	*Roseburia inulinivorans*, *Bacteroides uniformis*, and *Eubacterium rectale*	4	Rats	M	CUMS	Serum	↓ IL-1β; ↓ LPS; no effect: INF-γ	[67]

This table summarizes the results of studies examining the effects of psychobiotics on peripheral inflammation in animal models of depression. Studies investigating psychobiotics from the same genus are grouped together. The genera are arranged by the number of studies in which they have been investigated, with those most frequently used as psychobiotics listed first. The columns specify the genus of the psychobiotic, the strain used, animal subject in depression modeling (mice/rats), the sex of the animals (male (M) or female (F)), the procedure employed to induce depression (e.g., CSDS, CSDS, LPS-D), the sample used for inflammation-related parameter measurement (serum, plasma, colon, etc.) and the molecular changes observed after psychobiotic treatment, with ↑ denoting increases and ↓ denoting decreases relative to the depression animal model: CRS—chronic restraint stress; MS—maternal separation; CUMS—chronic unpredictable mild stress; SD—sleep deprivation; IM—immobilization; cFM—cultured fecal microbiota of patients with depression (cFM)-induced depression; LPS-D—lipopolysaccharide-induced depression; CUS—chronic unpredictable stress; CSDS—chronic social defeat stress; mALPS—murine alcohol-lipopolysaccharide model; IL—interleukin; INF-γ—interferon gamma; TNF-α—tumor necrosis factor-alpha; LPS—lipopolysaccharide; TLR—Toll-like receptor; MyD88—myeloid differentiation primary response 88; LBP—lipopolysaccharide binding protein; TGF-β1—transforming growth factor-β1; CXCL9—chemokine (C-X-C motif) ligand 9; CXCL10—chemokine (C-X-C motif) ligand 10; CCL2—chemokine (C-C motif) ligand 2; CD4 T cells—helper T cells; Treg cells—regulatory T cells; M1—pro-inflammatory macrophages; CRP—C-reactive protein; NLRP3—NOD-, LRR- and pyrin domain-containing protein 3 (NLRP3) inflammasome.

**Table 4:** The effects of psychobiotics on neuroinflammation in animal models of depression.

Psychobiotic		Treatment Duration (Weeks)	Animal	Sex (F/M)	Animal Model	Brain Region	Effect of Psychobiotic on Neuroinflammation in Depression Model	Ref.
Genus	Strain							
*Lactobacillus*	*Lactiplantibacillus plantarum* GOLDGUT-HNU082	8	Mice	M	CUMS	Brain	No effect: TNF-α; IL-1β; IL-6	[24]
	*Lactiplantibacillus plantarum* JYLP-326	3	Mice	M	CUMS	HIPP	↓ number of Iba1 positive cells ↓ TNF-α; ↓ IL-1β; ↓ IL-6; ↓ TLR4;	[25]
	*Lactiplantibacillus plantarum* P72	1	Mice	M	cFM	PFC	↑ IL-10	[26]
	*Lactiplantibacillus plantarum* WH021	4	Mice	M	LPS-D	Brain	↓ IL-1β; ↑ IL-10; ↓ Iba1 reactivity	[28]
	*Lactiplantibacillus plantrum* CR12	3	Mice	F	CUMS	HIPP	↓ expression of NLRP3 ↓ proportion of M1 microglia ↑ proportion of M2 microglia ↓ IL-6; ↑ IL-4 and IL-10 expression	[22]
	*Lactobacillus plantarum* GM11	3	Rats	M	CUMS	HIPP	↓ TNF-α No effect: IL-1β	[33]
	*Lacticaseibacillus rhamnosus* KY16	7	Mice	M	CUMS	Cortex	↓ TNF-α No effect: IL-10	[43]
	*Lactobacillus rhamnosus* HN001	6	Rats	M	CUMS	Brain	↓ TNF-α; ↓ IL-1β; ↓ IL-18; ↓ IL-6	[38]
	*Lactobacillus zhachilii* HBUAS52074T	4	Mice	M	CSDS	Brain	↓ INF-γ; ↑ TGF-β1	[39]
						HIPP	↓ TLR4; ↓ TNF-α; ↓ INF-γ; ↓ IL-6; ↓ Iba-1 reactivity	
						PFC	↓ TNF-α; ↓ INF-γ; ↓ IL-6; ↓ Iba-1 reactivity No effect: TLR4	
	*Lactocaseibacillus rhamnosus* zz-1	4	Mice	M	CUMS	HIPP	↓ TNF-α; ↓ IL-1β; ↓ IL-6	[44]
*Bifidobacterium*	*Bifidobacterium breve* CCFM1025	5	Mice	M	CUMS	HIPP	↓ IL-6; No effect: IL-1β; TNF-α; IL-17	[47]
						HIPP	↓ IL-1β; ↓ IL-6; ↑ IL-10; ↑ IL-22; ↓ Iba-1 reactivity	[48]
	*Bifidobacterium breve* M-16V	4.7 (33 days)	Mice	M	CSDS	PFC	↓ IL-1β; CCR2; Ym1 No effect: IL-6; TNF-α; CD68; Arg1 and CD206	[50]
						HIPP	↓ IL-1β; CCR2; Ym1 No effect: IL-6; TNF-α and CD68; Arg1; CD206	
	*Bifidobacterium lactis* HN019	6	Rats	M	CUMS	Brain	↓ TNF-α; ↓ IL-1β; ↓ IL-18; ↓ IL-6	[38]
	*Bifidobacterium longum* CECT 30763	6.6 (46 days)	Mice	M	CSDS	PFC	↓ TLR4; ↓ TLR5 No effect: TLR2; CCL2; TNF-α	[51]
						HIPP	↑ TLR2; ↓ TLR4; ↓ TLR5; ↓ CCL2; ↓ TNF-α	
						Striatum	↓ TLR2; ↓ TLR4; ↓ TLR5; ↓ CCL2	
	*Bifidobacterium longum* P77	1	Mice	M	IM	PFC	↓ TNF-α; ↓ IL-6; ↑ IL-10	[26]
					cFM	PFC	↑ IL-10	
Akkermansia	*Akkermansia muciniphila*	every 3 days for 2 weeks	Mice	F	mALPS	HIPP	↓ TNF-α; ↓ IL-1β; ↓ Iba-1 reactivity	[58]
*Bacillus*	*Bacillus coagulans* Unique IS-2	6	Rats	M/F	MS and CUMS	FC	Male: ↓ TNF-α; no effect CRP; IL-1β Female: no effect TNF-α; CRP; IL-1β	[19]
						HIPP	Male: no effect TNF-α; CRP Female: no effect TNF-α; CRP	
*Pediococcus*	*Pediococcus acidilactici* CCFM1344	4	Mice	No info.	CUMS	HIPP	↓ TLR4; ↓ TNF-α; ↓ IL-1β	[61]
*Weissella*	*Weissella paramesenteroides* WpK4	1.4 (10 days)	Mice	F	CRS	HIPP	↓ IL-1β; ↓ IL-6	[62]
*Lactobacillus*, *Bifidobacterium*, *Bacillus*	*B. coagulans* Unique IS-2 *L. plantarum* UBLP-40, *L. rhamnosus* UBLR-58, *B. lactis* UBBLa-70, *B. breve* UBBr-01, *B. infantis* UBBI-01	6	Rats	M/F	MS and CUMS	PFC	Male: ↓ TNF-α; no effect CRP Female: no effect TNF-α; CRP	[20]
						HIPP	Male: ↓ TNF-α; no effect CRP Female: ↓ TNF-α; no effect CRP	
*Lactobacillus*, *Bifidobacterium*	HN001 and HN019	6	Rats	M	CUMS	Brain	↓ TNF-α; ↓ IL-1β; ↓ IL-18; ↓ IL-6	[38]
	HN001 and HN019	6	Rats	M	CUMS	HIPP	↓ number of NLRP3 positive cells	[83]
						Cortex	↓ number of NLRP3 positive cells	
	P72+P77	1	Mice	M	cFM	PFC	↓ TNF-α; ↑ IL-10	[26]

This table summarizes the results of studies examining the effects of psychobiotics on neuroinflammation in animal models of depression. Studies investigating psychobiotics from the same genus are grouped together. The genera are arranged by the number of studies in which they have been investigated, with those most frequently used as psychobiotics listed first. The columns specify the genus of the psychobiotic, the strain used, animal subject in depression modeling (mice/rats), the sex of the animals (male (M) or female (F)), the procedure employed to induce depression (e.g., CSDS, IM, cFM), the sample used for neuroinflammation-related parameter measurement (PFC, HIPP, striatum, etc.) and the molecular changes observed after psychobiotic treatment, with ↑ denoting increases and ↓ denoting decreases relative to the depression animal model: CUMS—chronic unpredictable mild stress; cFM—cultured fecal microbiota of patients with depression (cFM)-induced depression; LPS-D—lipopolysaccharide-induced depression; CSDS—chronic social defeat stress; IM—immobilization; mALPS—murine alcohol-lipopolysaccharide model; MS—maternal separation; CRS—chronic restraint stress; HIPP—hippocampus; PFC—prefrontal cortex; FC—frontal cortex; TNF-α—tumor necrosis factor-alpha; IL—interleukin; Iba-1—ionized calcium-binding adaptor molecule 1; TLR—Toll-like receptor; NLRP3—NOD-, LRR- and pyrin domain-containing protein 3; M1—pro-inflammatory microglia; M2—anti-inflammatory microglia; INF-γ—interferon gamma; TGF-β1—transforming growth factor-β1; CCR2—chemokine receptor; Ym1—chitinase-like protein 3; CD68—cluster of differentiation 68; CCL2—chemokine (C-C motif) ligand 2; Arg1—arginase-1; CRP—C-reactive protein.

**Table 5:** The effects of psychobiotics on neurotransmission in animal models of depression.

Psychobiotic		Treatment Duration (Weeks)	Animal	Sex (F/M)	Animal Model	Sample	Effect of psychobiotic on Neurotransmission in Depression Model	Ref.
Genus	Strain							
*Lactobacillus*	*Lactobacillus fermentum* PS150	4	Rats	M	CUS	Whole brain	↑ 5-HT; No effect: DA; NE; GABA	[29]
	*Lactobacillus. helveticus* NS8	3.6 (25 days)	Rats	M	CRS	PFC	No effect: 5-HT; DA; NE	[30]
						HIPP	↑ 5-HT; ↑ NE; No effect: DA	
	*Lactiplantibacillus plantarum* GOLDGUT-HNU082	8	Mice	M	CUMS	Serum	↑ 5-HT; ↑ DA; ↑GABA; ↑ NE No effect: Ach; Glutamate	[24]
						Colon	↑ GABA No effect: 5-HT; DA; NE; Ach, Glutamate	
						Brain	↑ 5-HT; ↑ DA; ↑ NE No effect: GABA; Ach; Glutamate	
	*Lactiplantibacillus plantarum* JYLP-326	3	Mice	M	CUMS	HIPP	↑ 5-HT; ↑ 5-HT_1A_R; ↑ TPH2	[25]
	*Lactiplantibacillus plantarum* KLDS	5	Mice	M	CUMS	Serum	↑ DA; ↑ NE	[34]
						Brain	↑ 5-HT	
	*Lactiplantibacillus plantarum* P72	1	Mice	M	IM	PFC	↑ GABA; ↑ GABA_A_Rα1; ↑ GABA_A_Rα2; ↑ 5-HT; ↑ 5-HT_1A_R; ↑ 5-HT_1B_R	[26]
	*Lactiplantibacillus plantarum* P72				cFM	PFC	↑ GABA; ↑ GABA_A_Rα1; ↑ GABA_A_Rα2; ↑ 5-HT; ↑ 5-HT_1A_R; ↑ 5-HT_1B_R	
	*Lactiplantibacillus plantarum* R6-3	8	Mice	M	CUMS	Whole brain	↑ 5-HT; ↑ DA; ↑ NE	[27]
	*Lactobacillus plantarum* GM11	3	Rats	M	CUMS	Serum	↑ 5-HT	[33]
	*Lactobacillus plantarum* PS128	4	Mice	M	MS	PFC	↓ 5-HIAA; ↑ DA; No effect: 5-HT; DOPAC; HVA ↓ 5-HIAA:5-HT turnover ratio ↓ DOPAC:DA turnover ratio ↓ HVA:DA turnover ratio	[18]
						HIPP	No effect: 5-HT; 5-HIAA; DA; DOPAC; HVA	
						Striatum	No effect: 5-HT; 5-HIAA; DA; DOPAC; HVA	
	*Lactobacillus. plantarum* WLPL04	4	Mice	M	CRS	Serum	No effect: 5-HT	[36]
	*Lactobacillus paracasei* PS23	6	Mice	M	CORT-D	HIPP	↑ DA; ↑ 5-HT; No effect: DOPAC; 5-HIAA	[32]
						PFC	↑ DA; ↑ 5-HT; No effect: DOPAC; 5-HIAA	
						Striatum	↑ DA; ↑ 5-HT; No effect: DOPAC; 5-HIAA	
	*Lacticaseibacillus rhamnosus* TF318	4	Rats	M	CORT-D	Whole brain	↑ 5-HT; ↑ DA; No effect: NE	[74]
	*Lactobacillus rhamnosus* HN001	6	Rats	M	CUMS	Brain	↑ 5-HT; ↑ DA; ↓ DOPAC; ↑ NE	[38]
						Serum	↑ 5-HT; ↑ DA; ↑ NE	
	*Lactocaseibacillus rhamnosus* zz-1	4	Mice	M	CUMS	HIPP	↑ 5-HT	[44]
						Serum	↑ 5-HT	
	*Lactobacillus reuteri* ATG-F4	4	Mice	M	CUS	vHIPP	No effect: *Htr1a*; *Htr1b*; *Htr2a*	[89]
						mPFC	↓ *Htr1a*; No effect: *Htr1b*; *Htr2a*	
						BLA	No effect: *Htr1a*; *Htr1b*; *Htr2a*	
	*Lactobacillus zhachilii* HBUAS52074^T^	4	Mice	M	CSDS	Serum	↑ 5-HT	[39]
						Brain	↑ 5-HT	
*Bifidobacterium*	*Bifidobacterium breve* CCFM1025	5	Mice	M	CUMS	serum	↑ 5-HT	[47]
						HIPP	↑ 5-HT	
						PFC	↑ 5-HT	
	*Bifidobacterium lactis* HN019	6	Rats	M	CUMS	Brain	↑ 5-HT; ↑ DA; ↓ DOPAC; ↑ NE	[38]
						Serum	↑ 5-HT; ↑ DA; ↑ NE	
	*Bifidobacterium longum* CECT 30763	6.6 (46 days)	Mice	M	CSDS	Plasma	↓ DA	[51]
						Colon	↑ D1R; ↑ D2L; ↑ D2S; ↑ COMT No effect: MAOA	
						Ileum	No effect: D1R; D2L; D2S; COMT; MAOA	
						PFC	↑ D2L; ↑ MAOA; No effect: D1R and D2S, COMT	
						HIPP	↓ MAOA; No effect: D1R; D2L; D2S, COMT	
						Striatum	↑ COMT; No effect: D1R; D2L; D2S; MAOA	
	*Bifidobacterium longum* P77	1	Mice	M	IM	PFC	↑ GABA; ↑ GABA_A_Rα2; ↑ 5-HT; ↑ 5-HT_1B_R	[26]
					cFM	PFC	↑ GABA; ↑ GABA_A_Rα1; ↑ GABA_A_Rα2;	
*Lactococcus*	*Lactococcus lactis* WHH2078	4	Mice	M	CRS	HIPP	↑ 5-HT	[54]
	*Lactococcus lactis* WHH2078	5	Mice	M	CUMS	HIPP	↑ 5-HT	[17]
*Akkermansia*	*Akkermansia muciniphila*	every 3 days for 2 weeks	Mice	F	mALPS	HIPP	↑ 5-HT	[58]
	*Akkermansia muciniphila*	3	Mice	M	CRS	Serum	No effect: 5-HT; ↑ DA	[59]
*Bacillus*	*Bacillus coagulans* Unique IS-2	6	Rats	M/F	MS and CUMS	Frontal cortex	Male—no effect: DA; 5-HT; NE Female—no effect: DA; 5-HT; NE	[19]
	*Bacillus licheniformis*	4	Rats	M	CUMS	Whole Brain	↑ DA; ↑ Trp; ↑ GABA; ↓ Kyn; ↓ NE No effect: 5-HT; Glu	[60]
*Lactobacillus*, *Bifidobacterium*	HN001 and HN019	6	Rats	M	CUMS	Brain	↑ 5-HT; ↑ DA; ↓ DOPAC; ↑ NE	[38]
						Serum	↑ 5-HT; ↑ DA; ↑ NE	
	P72+P77	1	Mice	M	IM	Serum	↑ 5-HT; ↑ DA; ↑ NE	[26]
					cFM	PFC	↑ GABA; ↑ GABA_A_Rα1; ↑ GABA_A_Rα2; ↑ 5-HT; ↑ 5-HT_1B_R	
*Lactobacillus*, *Bifidobacterium*, *Bacillus*	*B. coagulans* Unique IS-2 *L. plantarum* UBLP-40, *L. rhamnosus* UBLR-58, *B. lactis* UBBLa-70, *B. breve* UBBr-01, *B. infantis* UBBI-01	6	Rats	M/F	MS and CUMS	FC	Male: ↓ Kyn; ↓ KA; No effect: 5-HT; DA; NE; Trp Female: ↓ Kyn; ↓ KA; No effect: 5-HT; DA; NE; Trp	[20]
*Lactobacillus*, *Lactococcus*, *Streptococcus*, *Bifidobacterium*	*L. helveticus* LA 102, *Bifidobacterium longum* LA 101, *Lactococcus lactis* LA 103, and *Streptococcus thermophilus* LA 104	5	Rats	M	MS	HIPP	No effect: DA; DOPAC; HVA; DOPAC/DA; HVA/DA; 5-HT; 5-HIAA; 5-HIAA/5-HT; NE	[65]
						Striatum	No effect: DA; 3-MT; HVA; DOPAC/DA; HVA/DA; 3-MT/DA; 5-HT; 5-HIAA; 5-HIAA/5-HT; NE ↑ DOPAC	

This table summarizes the results of studies examining the effects of psychobiotics on neurotransmission in animal models of depression. Studies investigating psychobiotics from the same genus are grouped together. The genera are arranged by the number of studies in which they have been investigated, with those most frequently used as psychobiotics listed first. The columns specify the genus of the psychobiotic, the strain used, animal subject in depression modeling (mice/rats), the sex of the animals (male (M) or female (F)), the procedure employed to induce depression (e.g., CUMS, IM, cFM), the sample used for neurotransmission-related parameter measurement (plasma, colon, ileum, etc.) and the molecular changes observed after psychobiotic treatment, with ↑ denoting increases and ↓ denoting decreases relative to the depression animal model: CUS—chronic unpredictable stress; CRS—chronic restraint stress; CUMS—chronic unpredictable mild stress; IM—immobilization; cFM—cultured fecal microbiota of patients with depression (cFM)-induced depression; MS—maternal separation; CORT-D—corticosterone-induced depression; CSDS—chronic social defeat stress; mALPS—murine alcohol-lipopolysaccharide model; PFC—prefrontal cortex; HIPP—hippocampus; vHIPP—ventral hippocampus; mPFC—medial prefrontal cortex; BLA-basolateral amygdala; FC—frontal cortex; 5-HT—5-hydroxytryptamine; DA—dopamine; NE—norepinephrine; GABA—gamma-aminobutyric acid; Ach—acetylcholine; 5-HT_1A_R—serotonin 1A receptor; TPH2— tryptophan hydroxylase 2; GABA_A_Rα1—gamma-aminobutyric acid type A receptor subunit alpha1; GABA_A_Rα2—gamma-aminobutyric acid type A receptor subunit alpha2; 5-HT_1B_R—serotonin 1B receptor; 5-HIAA—5-hydroxyindoleacetic acid; DOPAC—3,4-dihydroxyphenylacetic acid; HVA—homovanillic acid; *Htr1a*, *Htr1b*, *Htr2a*—genes that encode 5-HT1A, 5-HT1B and 5-HT2A serotonin receptors; D1R—D1 dopamine receptor; D2L and D2S—two isoforms of D2 dopamine receptors; COMT—catechol-O-methyltransferase; MAOA—monoamine oxidase A; Trp—tryptophan; Kyn—kynurenine; Glu—glutamine; KA—kynrenic acid; 3-MT—3-methoxytyramine.

## Data Availability

No new data were created or analyzed in this study. Data sharing is not applicable to this article.

## Supplementary Materials

The following supporting information can be downloaded at: https://www.mdpi.com/article/10.3390/pharmaceutics18010140/s1, Table S1: A detailed overview of the effects of psychobiotics on depression- and anxiety-like behaviors in animal models of depression.

## Abbreviations

The following abbreviations are used in this manuscript:

HPA	Hypothalamic-pituitary adrenal axis
CNS	Central nervous system
5-HT	Serotonin
GABA	Gamma-aminobutyric acid
DA	Dopamine
NA	Noradrenaline
SCFAs	Short-chain fatty acids
MGBA	Microbiota-gut-brain axis
Trp	Tryptophan
CUMS	Chronic unpredictable mild stress
M	Male
F	Female
IM	Immobilization stress
cFM	Cultured fecal microbiota of patients with depression
LPS	Lipopolysaccharide
CSDS	Chronic social defeat stress
ACTH	Adrenocorticotrophic hormone
MS	Maternal separation
CORT	Corticosterone
SD	Sleep deprivation
CRS	Chronic restraint stress
CUS	Chronic unpredictable stress
mALPS	Murine alcohol-lipopolysaccharide model
TST	Tail suspension test
SPT	Sucrose preference test
OFT	Open field test
EPM	Elevated plus maze
EZM	Elevated zero maze
FST	Forced swim test
LDB	Light-dark box
MBT	Marble burying test
SIT	Social interaction test
Nr3c1	Nuclear receptor subfamily 3 group C member 1
CRH	Corticotrophin-releasing hormone
HIPP	Hippocampus
GR	Glucocorticoid receptor
PFC	Prefrontal cortex
IL	Interleukin
TNF-α	Tumor necrosis factor-alpha
INF-γ	Interferon gamma
CXCL9	Chemokine (C-X-C motif) ligand 9
CXCL10	Chemokine (C-X-C motif) ligand 10
CCL2	Chemokine (C-C motif) ligand 2
TLR	Toll-like receptor
MyD88	Myeloid differentiation primary response 88
LBP	Lipopolysaccharide binding protein
TGF-β1	Transforming growth factor-β1
NLRP3	NOD-, LRR- and pyrin domain-containing protein 3
CRP	C-reactive protein
QUIN	Quinolinic acid
vHIPP	Ventral hippocampus
mPFC	Medial prefrontal cortex
BLA	Basolateral amygdala
D1R	D1 dopamine receptor
D2L	D2 dopamine receptors, isoform L
D2S	D2 dopamine receptors, isoform S
COMT	Catechol-O-methyltransferase
MAOA	Monoamine oxidase A
GABA_A_Rα1	Gamma-aminobutyric acid type A receptor subunit alpha1
GABA_A_Rα2	Gamma-aminobutyric acid type A receptor subunit alpha2
5-HT_1A_R	Serotonin 1A receptor
5-HT_1B_R	Serotonin 1B receptor
NE	Norepinephrine
Ach	Acetylcholine
Glu	Glutamine
Kyn	Kynurenine
DOPAC	3,4-dihydroxyphenylacetic acid
5-HIAA	5-hydroxyindoleacetic acid
HVA	Homovanillic acid
KA	Kynrenic acid
3-MT	3-methoxytyramine
5-HTT	5-HT- transporter
5-HTP	5-hydroxytryptophan
BBB	Blood–brain barrier
TPH1	Trp hydroxylase 1
PV	Parvalbumin
ILA	Indole-3-lactic acid
KYNA	Kynurenic acid
