# Supplementary material for: Current Evidence from Animal Models on Molecular Changes Underlying Antidepressant Effects of Psychobiotics
Source: Pharmaceutics. 2026 Jan 22;18(1):140. doi: 10.3390/pharmaceutics18010140 (PMC12844937; doi:10.3390/pharmaceutics18010140)
Supplement: Supplementary file 1 [file pharmaceutics-18-00140-s001.zip › pharmaceutics-4047925-supplementary.pdf]

Table S1. A detailed overview of the effects of psychobiotics on depression- and anxiety-like behaviors in animal models of depression.

Psychobiotic		Treatment duration (weeks)	Animal	Sex (M / F)	Animal model	Behavioral test								Ref
						Depression behavior			Anxiety behavior					
Genus	Strain					TST (immobility time)	SPT (sucrose preference)	FST (immobility time)	SIT	OFT	EPM	LDB	MBT (No of buried marbles)	
Lactobacillus	*Lactiplantibacillus plantarum* CR12	3	Mice	F	CUMS	↓	↑	↓						[22]
	Lactiplantibacillus plantarum D-9	2	Mice	M	CUMS	↓	↑	↓		↑ time in central area	↑ time spent in open arms; ↑ number of entries in open arms			[23]
	Lactiplantibacillus plantarum GOLDGUT-HNU082	8	Mice	M	CUMS	↓	↑	↓		↑ time in central area	No effect: time spent in open arms			[24]
	Lactiplantibacillus plantarum JYLP-326	3	Mice	M	CUMS	↓	↑			↑ time in central area; ↑ number of crossings and total distance moved				[25]
	Lactiplantibacillus plantarum P72	1	Mice	M	IM	↓				↑ total distance traveled ; ↑ distance traveled in central area; ↑ time in central area	↑ open arm entries; ↑ time spent in open arms			[26]
	Lactiplantibacillus plantarum P72				cFM	↓				↑ total distance traveled; ↑ distance traveled in central area¸↑ time in central area	↑ open arm entries; ↑ time spent in open arms			
	Lactiplantibacillus plantarum R6-3	8	Mice	M	CUMS	↓	↑	↓						[27]
	Lactiplantibacillus plantarum WH021	4	Mice	M	LPS-D	↓				↑ distance traveled in central area; ↑ total distance traveled				[28]

	Lactobacillus fermentum PS150	4	Rats	M	CUS			↓		↓ time spent in closed arm; ↑ exploratory duration and ↑ number of rearing	[29]
	Lactobacillus helveticus NS8	3.6 (25 days)	Rats	M	CRS		↑		↑ distance travelled in central area; No effect: time spent in central area	↑ time spent in open arms; No effect: entries in open arms	[30]
	Lactobacillus paracasei Lpc-37	5	Mice	M	CRS			↑ swimming time; ↓ immobility time			[31]
	Lactobacillus plantarum LP12407								↑ time spent in central area		
	Lactobacillus paracasei PS23	6	Mice	M	CORT-D		↑	↓	↑ total distance trav-eled¸↑ time in central area; ↑ number of entries into the center area		[32]
	Lactobacillus plantarum GM11	3	Rats	M	CUMS		↑	↓	No effect	No effect	[33]
	Lactobacillus plantarum KLDS1.0386	5	Mice	M	CUMS	↓	↑	↓	↑ total distance traveled; ↓ immobility time		[34]
	Lactobacillus plantarum MTCC 9510	4	Mice	M	CUMS and SD	↓		↓		↑ time spent in open arms (EZM)	[35]
	Lactobacillus plantarum PS128	4	Mice	M	MS		↑	↓	↑ total distance traveled, No effect: time in central area	No effect: time spent in close arms	[18]
	Lactobacillus plantarum WLPL04	4	Mice	M	CRS			↓	↑ time in central area;	↑ number of entries and time spent in open arms	[36]
	Lactobacillus rhamnosus GG (ATCC 53103)	2	Rats	M	CUMS		No effect	↓			[37]

	Lactobacillus rhamnosus HN001	6	Rats	M	CUMS					↑ crossing numbers; ↑ numbers of standing	↑ total distance traveled; ↑ time in the close arms		[38]
	Lactobacillus zhachilii HBUAS52074T	4	Mice	M	CSDS	↓	↑		↑ social interaction ratio; ↑ time in interaction zone	↑ distance traveled in central area; ↑ time in central area; ↑ number of entries into center			[39]
	Lacticaseibacillus casei IDCC 3451	10	Mice	M	CUMS	↓	↑	↓		↑ distance traveled in central area; ↑ time in central area	↑ time in the open areas, ↑ open entries (EZM)		[40]
	Lacticaseibacillus rhamnosus IDCC3201	9	Mice	M	CUMS			No effect			↑ time spent in open arms;<br>↓ time spent in closed arms		[41]
	Lacticaseibacillus rhamnosus JB-1	8	Rats	M	CUMS						↑ time spent in open arms		[42]
	Lacticaseibacillus rhamnosus KY16	7	Mice	M	CUMS		↑	↓		↑ total distance traveled	↑ open arm entries		[43]
	Lactocaseibacillus rhamnosus zz-1	4	Mice	M	CUMS	↓	↑				↑ time spent in open arms		[44]
Bifidobacterium	*Bifidobacterium adolescentis* NGB329	8	Rats	M	CUMS		↑				↑ time spent in open arms	↓	[45]
	Bifidobacterium breve Bre1025	4	Mice	M	CUMS	↓		↓		↑ time in central area			[46]
	Bifidobacterium breve CCFM1025	5	Mice	M	CUMS	↓	↑	↓		↑ time in central area	↑ time spent in the open arms		[47]

	Bifidobacterium breve CCFM1025	5	Mice	M	CUMS			↓		↑ time in central area	↑ time spent in open arms	↑ delay time of entering the dark box	[48]
	Bifidobacterium breve CCFM1025	6	Mice	M	CUMS	↓		↓		↑ time in central area	↑ time in open arms		[49]
	Bifidobacterium breve M-16V	4.7 (33 days)	Mice	M	CSDS				↑ the time in interaction zone; ↓ the time in corner zones				[50]
	Bifidobacterium infantis	7	Rats	M/F	MS			M – no effect, F - ↑ swimming time					[21]
	Bifidobacterium lactis HN019	6	Rats	M	CUMS					↑ crossing numbers; ↑ numbers of standing	↑ total distance traveled; ↑ time in the close arms		[38]
	Bifidobacterium longum CECT 30763	6.6 (46 days)	Mice	M	CSDS	No effect: time to stop moving	↑	↓		No effect: numbers of entries into the central area		↓ latency in moving to the light area	[51]

	Bifidobacterium longum P77	1	Mice	M	IM	↓			↑ total distance traveled ; ↑ distance traveled in central area; ↑ time in central area	↑ open arm entries; ↑ time spent in open arms	[26]
	Bifidobacterium longum P77				cFM	↓			↑ distance traveled in central area; ↑ time in central area	↑ open arm entries; ↑ time spent in open arms	[26]
	Bifidobacterium pseudocatenulatum CECT 7765	3	Mice	M	MS					↑ time spent in open arms	[52]
Lactococcus	*Lactococcus lactis subsp. cremoris* LL95	1	Mice	M	LPS			↓			[53]
	Lactococcus lactis WHH2078	5	Mice	M	CUMS	↓	↑	↓	↑ time in central area; ↑ travel distance		[17]
	Lactococcus lactis WHH2078	4	Mice	M	CRS	↓	↑	↓	↑ time in central area		[54]
	Lactococcus lactis ZFM559	5	Mice	M	CUMS	↓	↑		↑ total distance traveled		[55]
Faecalibacterium	*Faecalibacterium prausnitzii* ATCC 27766	6	Rats	M	CUMS+ ACTH		↑	↓	No effect: total distance traveled	No effect: open arm entries, time spent in open arms, total entries in closed arms	[56]
	Faecalibacterium prausnitzii ATCC 27766	4	Rats	M	CUMS			↓	↓ latency to the center, No effect: entries and time spent in the central area	↑ number of entries and the time spent in the open arms	[57]
Akkermansia	*Akkermansia muciniphila*	every 3 days for 2 weeks	Mice	F	mALPS	↓		↓	↑ total distance moved; ↓ time in peripheral zone		[58]
	Akkermansia muciniphila	3	Mice	M	CRS	↓		↓	↑ total distance moved		[59]

Bacillus	*Bacillus coagulans Unique IS-2*	6	Rats	M/F	MS and CUMS		↑	↓		↑ number of entries and time spent in the open arms		[19]
	Bacillus licheniformis	4	Rats	M	CUMS			↓		↑ time spent in open arms		[60]
Pediococcus	*Pediococcus acidilactici* CCFM1344	4	Mice	No info.	CUMS	↓			↑ time in central area	↑ time spent in open arms		[61]
Weissella	*Weissella paramesenteroides* WpK4	1.4<br>(10 days)	Mice	F	CRS	↓				↑ time in open arms		[62]
Phocaeicola	*Phocaeicola vulgatus* NGB218	8	Rats	M	CUMS		↑			↑ time spent in open arms; ↓ time spent in closed arms; ↓ number of entries in closed arms	↓	[63]
Lactobacillus, Bifidobacterium	HN001 and HN019	6	Rats	M	CUMS				↑ crossing numbers; ↑ numbers of standing	↑ total distance traveled; ↑ time in the close arms		[38]
Lactobacillus, Streptococcus	*Lactobacillus plantarum* NBIMCC 8767 + *Streptococcus thermophilus* NBIMCC 8258	2	Rats	M	CUMS		↑	↓				[64]
Bacillus,<br>*Lactobacillus,*<br>*Bifidobacterium*	Multi-strain probiotic formulation: Bacillus coagulans Unique IS-2, Lactiplantibacillus plantarum UBLP-40, Lactobacillus rhamnosus UBLR-58, Bifidobacterium lactis UBBLa-70, Bifidobacterium breve UBBr-01, Bifidobacterium infantis UBBI-01	6	Rats	M/F	MS and CUMS		↑	↓	No effect: total distance traveled	↑ number of entries and the time spent in the open arms		[20]

Lactobacillus, *Bifidobacterium*, *Lactococcus*, *Streptococcus*	Multi-strain probiotic formulation: *Lactobacillus helveticus* LA 102, *Bifidobacterium longum* LA 101, *Lactococcus lactis* LA 103, *Streptococcus thermophilus* LA 104	5	Rats	M	MS			↓	↑ time in central area		↑ exit attemp ts	[65]
Lactobacillus, *Bifidobacterium*	Multi-strain probiotic formulation: *Lactobacillus plantarum* ATCC 793, *Bifidobacterium longum* ATCC 15707	11	Mice	M	CUS			↓	↑ time in central area			[66]
Lactobacillus, *Bifidobacterium*	P72+P77	1	Mice	M	IM	↓			↑ total distance traveled ; ↑ distance traveled in central area; ↑ time in central area	↑ open arm entries; ↑ time spent in open arms		[26]
					cFM	↓			↑ distance traveled in central area; ↑ time in central area	↑ open arm entries; ↑ time spent in open arms		
Roseburia, *Bacteroides*, *Eubacterium*	Multi-strain probiotic: *Roseburia inulinivorans*, *Bacteroides uniformis*, and *Eubacterium rectale*	4	Rats	M	CUMS			↓	↑ time in central area	↑ open arm entries; ↑ time spent in open arms		[67]

This Table summarizes the results of studies examining the effects of psychobiotics on depression- and anxiety-like behaviors in animal models of depression. Studies investigating psychobiotics from the same genus are grouped together. The genera are arranged according to the number of studies in which they were investigated, with those genera including bacterial strains most frequently used as psychobiotics listed first. The columns specify: the genus of the psychobiotic, the strain used, the animal subject in depression modeling (mice/rats), the sex of the animals (male (M) or female (F)), the procedure employed to induce depression (CUMS, IM, cFM, etc.), and behavioral changes after treatment with psychobiotics. Depression-related behaviors include immobility time in the tail suspension test (TST), sucrose preference in the sucrose preference test (SPT); immobility time in the forced swim test (FST), and parameters from the social interaction test (SIT), specified in the

corresponding fields. Anxiety-related behaviors include parameters from the open field test (OFT), elevated plus maze test (EPM), elevated zero maze test (EZM), light-dark box test (LDB), and marble burying test (MBT), specified in the corresponding fields. In the Table, ↑ denotes increases and ↓ denotes decreases relative to the depression animal model. Abbreviations: CUMS – chronic unpredictable mild stress; IM – immobilization stress; cFM – cultured fecal microbiota of patients with depression (cFM)-induced depression; LPS-D – lipopolysaccharide-induced depression; CSDS – chronic social defeat stress; ACTH – adrenocorticotropic hormone; MS – maternal separation; CORT-D – corticosterone-induced depression; SD – sleep deprivation; CRS – chronic restraint stress; CUS – chronic unpredictable stress; mALPS – murine alcohol-lipopolysaccharide model.
